# Strategic identity signaling in heterogeneous networks

**DOI:** 10.1073/pnas.2117898119

**Published:** 2022-03-03

**Authors:** Tamara van der Does, Mirta Galesic, Zackary Okun Dunivin, Paul E. Smaldino

**Affiliations:** ^a^Santa Fe Institute, Santa Fe, NM 87501;; ^b^Complexity Science Hub Vienna, 1080 Vienna, Austria;; ^c^Vermont Complex Systems Center, University of Vermont, Burlington, VT 05405;; ^d^Center for Complex Networks and Systems Research, Luddy School of Informatics, Computer Science, and Engineering, Indiana University, Bloomington, IN 47408;; ^e^Department of Sociology, Indiana University, Bloomington, IN 47405;; ^f^Department of Cognitive and Information Sciences, University of California, Merced, CA 95343

**Keywords:** covert signaling, political identity, pragmatics, networks, Twitter

## Abstract

Individuals often signal identity information to facilitate assortment with partners who are likely to share norms, values, and goals. However, individuals may also be incentivized to encrypt their identity signals to avoid detection by dissimilar receivers, particularly when such detection is costly. Using mathematical modeling, this idea has previously been formalized into a theory of covert signaling. In this paper, we provide an empirical test of the theory of covert signaling in the context of political identity signaling surrounding the 2020 US presidential elections. To identify likely covert and overt signals on Twitter, we use methods relying on differences in detection between ingroup and outgroup receivers. We strengthen our experimental predictions with additional mathematical modeling and examine the usage of selected covert and overt tweets in a behavioral experiment. We find that participants strategically adjust their signaling behavior in response to the political constitution of their audiences. These results support our predictions and point to opportunities for further theoretical development. Our findings have implications for our understanding of political communication, social identity, pragmatics, hate speech, and the maintenance of cooperation in diverse populations.

## Significance

Much of online conversation today consists of signaling one’s political identity. Although many signals are obvious to everyone, others are covert, recognizable to one’s ingroup while obscured from the outgroup. This type of covert identity signaling is critical for collaborations in a diverse society, but measuring covert signals has been difficult, slowing down theoretical development. We develop a method to detect covert and overt signals in tweets posted before the 2020 US presidential election and use a behavioral experiment to test predictions of a mathematical theory of covert signaling. Our results show that covert political signaling is more common when the perceived audience is politically diverse and open doors to a better understanding of communication in politically polarized societies.

Individuals constantly emit signals of their identity, consciously and unconsciously, informing others about the sort of person they are. Identity signals are any components of communication that inform receivers of the signaler’s membership (or nonmembership) in a subset of individuals (1–3). Such subsets can reflect strong social boundaries, such as “Republican” or “Democrat” in the United States, or reflect subtler intragroup variations, such as differences among Democrats regarding government regulations. In large, multicultural nations like the United States, identities such as Republican or Democrat can serve to organize like-minded communities or coalitions (4–7). Although the specific style of communication may vary with cultural context (8), identity signaling serves a key social function by enabling individuals to rapidly characterize others as similar or dissimilar (2, 3, 9). Finding similar others has many proximate psychological benefits, such as better mental health (10) and the security that results from a stronger sense of group identity (11, 12). Our emphasis here is on the role of identity signaling to facilitate social assortment: preferentially interacting with similar individuals and reaping the benefits of coordinating on norms, goals, and values (2, 3, 9, 13–15).

Identity signaling is especially important in vast and diverse social communities, in which little can be assumed about strangers in the absence of identity information. This type of scenario is made all the more common in the digital age (16). In recent years, online social media has both expanded the pool of potential partners and enabled easier formation of communities across traditional, social, and geographic boundaries. This presents new challenges and opportunities for signalers to successfully find niche communities (17, 18). On the one hand, large online communities have arisen dedicated to worldviews that are otherwise rare in most local communities. An individual expressing a viewpoint that is rare in their locality can nevertheless become part of a flourishing, geographically dilute collective. On the other hand, online signaling also carries new risks that come from expanding one’s audience far beyond one’s local social network, sometimes without the signaler’s knowledge (e.g., ref. 19).

Given the social importance of political identity in the United States and other countries (5, 20), we expect much identity signaling to be about political views and related coalitional affiliations. Political views are often expressed on social media using obvious signals like slogans, partisan memes, and other declarations of partisanship. However, the United States is also highly polarized (5, 6), and obvious political signals are not always advisable. Partisans often hold deeply negative feelings toward members of groups perceived as opposed or even simply different to their own (21–24). Signaling one’s political affiliation to outgroup members can therefore be costly, with costs ranging from the loss of social standing or relationship opportunities to the loss of an employment (25) or even becoming the victim of violence (26). For example, Van Duyn (27) documents a group of anti-Trump women in rural Texas who met in secret to discuss politics because they feared negative consequences for their business or marriages if their views became known. Exactly who is considered a member of one’s outgroup also varies over time and context. In the context of political identities, debates during US presidential primaries tend to be between members of the same political party, and so, a perceived outgroup may be copartisans that support different candidates or policy goals. During national presidential elections, cross-partisan differences become more salient. In both cases, the assortative benefits of overt identity signaling must be weighed against the potential costs of being identified by outgroup individuals in situations in which identification has consequences.

Overt, unambiguous signals of identity are useful when individuals can sufficiently benefit from their role in supporting positive assortment—preferentially interacting with similar others. A wide literature on social tags and ethnic markers has documented and modeled the utility and likely emergence of such signals for this purpose (9, 13, 28–31). The benefits of overt identity signaling, however, must sufficiently outweigh any risks that come from alienating others or revealing oneself to be misaligned with their interests. If those risks and their associated costs are high enough, we should expect cultural or psychological processes (such as cultural evolution or strategic decision-making) to favor subtler signaling strategies that encode information in such a way that it is detectable only by those who share relevant worldviews.

We refer to identity signals that are accurately received by their intended audience but obscured when received by others as covert signals (3, 32, 33). Covert signals allow individuals to reap at least some benefit from being identified by similar others, when possible, while simultaneously avoiding the costs associated with detection by dissimilar others. Covert signals work because communication often contains multiple, simultaneous layers of meaning, which are not all equally apparent to all receivers. A receiver’s background knowledge and perception of context affects whether and how those layers will be revealed (34–37). Individuals should, therefore, adjust their patterns of communication, based not only on their intended audience but also on the likely third parties that will perceive those communications (37–39). Loury (39) captures the essence of the idea: “If the significance of some words as signals of belief is known only to ‘insiders,’ their use in public allows the speaker to convey a reassuring message to some listener—‘I share your values’—without alarming the others.” A well-documented example is the routine remarks made by former US president George W. Bush concerning his opposition to the 1857 Dred Scott Supreme Court decision. While it might seem unnoteworthy to oppose a judicial decision that upheld slavery, the mention was seen by many evangelical conservatives as morally analogous to the 1973 Roe v. Wade case that upheld the right to abortion and so subtly communicated to these audiences the president’s commitment to overturning that decision (40).

Recently, Smaldino and colleagues have developed a theory of covert signaling, using formal mathematical and agent-based models of cultural evolution to examine the circumstances under which overt or covert identity-signaling strategies should be favored (32, 33). The theory of covert signaling provides a formalism for identity signaling in the context of third-party receivers and describes how signalers should communicate based on their likely audiences and the consequences for both successful and failed communication. The models derived from this theory make general predictions about strategies for identity signaling related to both the ability of individuals to preferentially assort with similar others and the costs of failing to assort accordingly. Covert signaling can achieve higher payoffs than overt signaling when individuals are likely to have interactions with dissimilar individuals and when those interactions incur high costs once the dissimilarity is revealed. The theory of covert signaling is consistent with a number of common signaling domains, including the use of humor as an encrypted signal of similarity (41, 42), the use of fashion to subtly signal insider status (43), political dog whistles (44, 45), and signals used by LGBTQ+ individuals (46, 47) or political dissidents (48–50) to assort without detection. It is also consistent with the fact that signals of political identity need not be obviously political in nature, as reliable associations with certain products and activities may be used as heuristics to differentiate partisans (51–53).

**Fig. 1.**
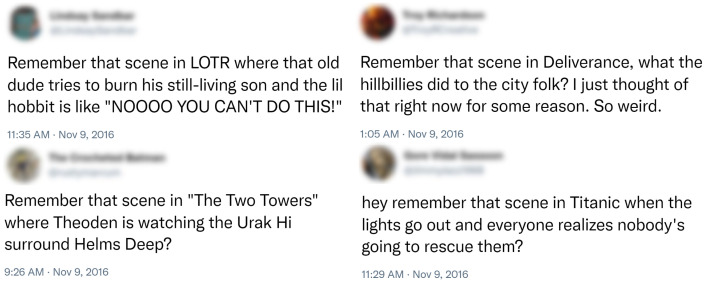
Potentially covert tweets related to the 2016 US presidential election. To understand each tweet, the reader must be familiar with both the ingroup conversations about the relevant political events as well as the cultural artifacts being referenced.

It is likely that a great deal of online speech is covert, especially on social media platforms on which users can be personally identified, such as Twitter. Although other social media sites have more users than Twitter (54), Twitter is a particularly important forum for public discourse on current events and as such is valuable for studying covert identity signals that are likely to be both relevant and visible to diverse audiences. For example, the strategy of “subtweeting” is well documented and refers to online communications that are interpretable only to individuals who have relevant information that is not provided in the communication itself (55). As another example, a search for tweets containing the phrase “remember that scene” sent on November 9, 2016 (the day after Donald Trump was elected as US president) returned a number of candidate covert tweets concerning feelings about the election results, many from users unhappy with the outcome (Fig. 1). Each of these requires background knowledge about the cultural artifact (i.e., film) being referenced as well as an understanding of recent political events, as the relevant contextual backdrop for interpreting the analogy implied by those references.

Our data were collected during an especially salient period for political identity signaling on Twitter: the 2020 COVID-19 pandemic, in which millions of people were restricted from gathering and communicating in person, making online engagement one of the principal ways to interact with other humans. Moreover, our tweets were collected and assessed in the wake of many high-profile sociopolitical events in the United States, including the Black Lives Matter protests following the murder of George Floyd, the confirmation of Supreme Court Justice Amy Coney Barrett, and the 2020 presidential election. Opinions on these events were often polarized, making expressions of those opinions into signals of political identity.

**Fig. 2.**
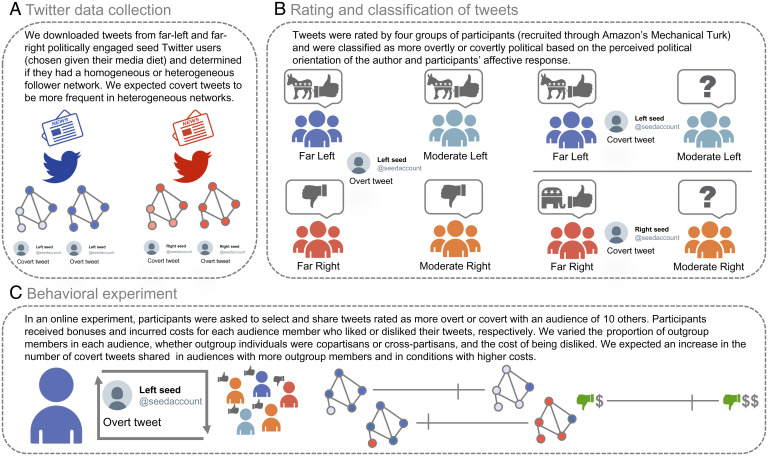
Empirical process to test the theory of covert signaling: (*A*) selecting Twitter users who might be more likely to use covert or overt political speech and downloading their tweets, (*B*) rating of tweets on two dimensions and using these ratings to select a subset of covert or overt tweets, and (*C*) conducting a behavioral experiment with the selected covert and over tweets to test how people use them to communicate their political belief.

While they are likely to be common, covert signals are also inherently challenging to study empirically because, by definition, they require insider knowledge to be detected. In this paper, we introduce a theoretically motivated measurement of covertness, focusing on identity signals in the context of political speech online. Essentially, covertness was measured in terms of how people from ingroup and outgroup political groups perceived different tweets. On Twitter, cross-partisan followers are rare (56), and thus, we focused on differences between copartisan radicals and moderates. We downloaded tweets from politically engaged Twitter users with heterogeneous follower networks, thus increasing our chances to collect tweets with some covert political identity signaling according to the theory (Fig. 2*A*). Then, we asked ingroup and outgroup members to guess the political identity of the tweet author and to report their affective responses to the tweet. Tweets were considered to be more likely to serve as covert identity signals if there was a large difference in responses of ingroup and outgroup raters (Fig. 2*B*).

Our paper constitutes a direct empirical test of the theory of covert signaling. Based on this theory, we predict that covert signaling will be more prevalent among 1) individuals in more heterogeneous communities or individuals with minority status and 2) individuals who face higher costs from being recognized as dissimilar. We derive a simple mathematical model of our experimental design, which yields more precise predictions concerning the relationships between covert signaling, the frequency of outgroup members in the audience, and the cost of being disliked. We test these predictions in a behavioral experiment in which participants select from a set of tweets that contain either overt or covert political signals to communicate with and be evaluated by an audience of varying partisanship (Fig. 2*C*). We compare signaling strategies when the outgroup audience consists of copartisan members (more or less radical) and cross-partisan members (left or right). Although previous studies have also considered the use of covert or encrypted signals (41, 43, 47, 57), our study tests predictions derived from a formal model with relatively unambiguous predictions and a clear scope of applicability (sensu ref. 58). By doing so, we can show where the existing theory fits the real world and where we need to direct our future efforts to refine the theory. Our empirical pipeline (Fig. 2) is described in more detail in the *Materials and Methods*.

## Results

### Audience Response to Overt and Covert Signals.

**Fig. 3.**
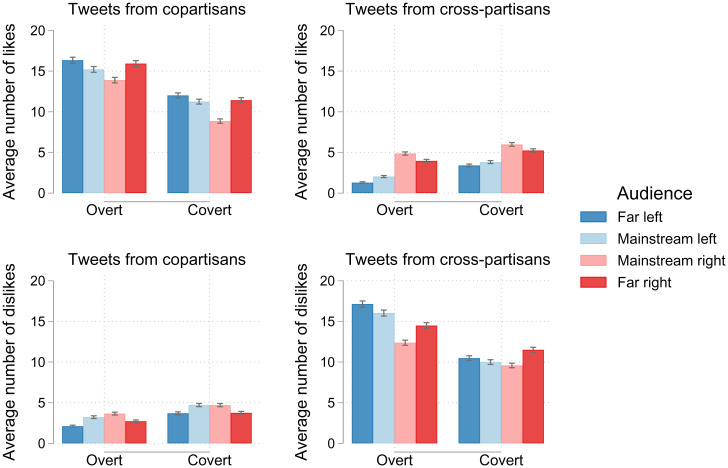
Average number of overt and covert tweets that were liked by co- and cross-partisan audience members (*n* = 481). Error bars represent the SE of the mean, assuming a Poisson distribution. Figs. 3–6 are created in STATA with the *cleanplots* scheme (59) and the *palettes* package (60).

Audience members in the behavioral experiment liked and disliked covert and overt tweets in line with our definitions of covert and overt signaling. At the same time, as might be expected from a comparison between theoretical modeling and empirical reality, participant behavior was more nuanced than our original theoretical assumptions. Overall, as shown in Fig. 3, all groups tended to like tweets from their own side of the political spectrum (copartisans) and tended to dislike tweets from the opposite side (cross-partisans). There were also marked differences between moderates and radicals. Moderates on average liked fewer tweets from copartisans and disliked fewer tweets from cross-partisans compared to radicals. Covert tweets were liked less often by copartisans and disliked less often by cross-partisans compared to overt tweets. Unlike simplified assumptions in prior theoretical models (32, 33), we find here that using covert tweets only decreases, but does not completely prevent, dislikes from the outgroup. Regardless, the best strategy for maximizing likes and reducing dislikes remains consistent with the expectations from the theory of covert signaling: sharing more covert tweets as the size of the outgroup increases and when the cost of dislikes is high. With this information in hand, we derive more precise predictions for the advantage of covert compared to overt signaling in a mathematical model.

### Mathematical Predictions for Signaling Strategies with Varying Outgroup Sizes and Costs.

In order to better interpret the results of our experiment, we developed a simple mathematical model in which we consider the relative expected payoffs to pure strategies of covert or overt signaling. The model allows us to make predictions under the assumption of rational behavior, though the source of that rationality could stem from either individual strategizing or population-level selection on optimal signaling strategies (61).

In an audience of size *n*, a proportion, *d*, will be members of the outgroup, and the rest will be members of the ingroup. We assume that overt signals are received (that is, the identity signal denoting similarity or dissimilarity is perceived) by all audience members with probability, *R*, and that covert signals are received by ingroup members with a probability $r_{I}<R$ and are perceived by outgroup members with probability *r_O_* < *r_I_*. Audience members who perceive similarity will like the signal, conferring a benefit, *b*, on the signaler, while audience members who perceive dissimilarity will dislike the signal, imposing a cost, *c*, on the signaler. These costs and benefits are added to the baseline benefit for participation, *w*_0_.

The expected payoff to an overt signaler is therefore the following:

$$W_{O}=w_{0}+R\left[(1-d)nb-\mathrm{dnc}\right].$$

Similarly, the expected payoff to a covert signaler is the following:

$$W_{C}=w_{0}+r_{I}(1-d)nb-r_{O}dnc.$$

Covert signalers avoid some of the cost of being disliked but also receive fewer liking benefits. The relative advantage of covert signaling depends heavily on the proportion of outgroup members in the audience, as well as the signal efficiency of both overt and covert signals.

We calibrated the model to our experiment by setting *n* = 10, b=1, and allowing the cost to vary so c={0.5,1}, reflecting the low- and high-cost conditions, respectively. Our values for receiving probabilities (*R* = 0.8, $r_{I}=0.6$, and $r_{O}=0.5$) were estimated from our experimental data by approximating the average proportion of covert and overt tweets liked and disliked by co- and cross-partisans (Fig. 3). Note that this differs from the simplified assumption in prior models (32, 33) that *r_O_* = 0, indicating the importance of testing these models empirically.

**Fig. 4.**
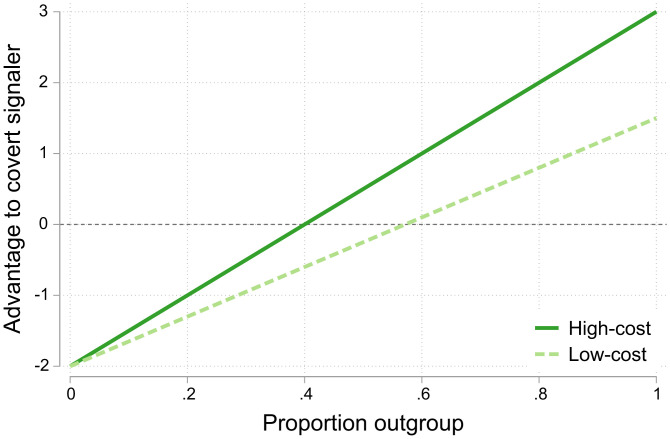
Difference in expected payoff between overt and covert signaling as a function of the outgroup proportion of the audience, *d*. Here, *n* = 10, *b* = 1, *R* = 0.8, $r_{I}=0.6$, and $r_{O}=0.5$. The costs are *c* = 1 in the high-cost condition (dark green) and *c* = 0.5 in the low-cost condition (light green).

Fig. 4 shows the relative payoff advantage to covert signaling (in cents) for experimental values. The figure shows that covert signaling is favored when the audience is composed primarily of outgroup members, and overt signaling is favored otherwise. Although we also see differences between the cost conditions, these manifest primarily with higher proportions of outgroup members, and the advantage to covert signaling under high cost is quite small relative to the difference between small and large values of *d*. We therefore expect a smaller effect of cost compared to outgroup size in our experiment. It is also worth noting that this simplified model focuses on pure strategies, predicting a total switch from overt to covert signaling as the audience reaches a critical threshold of outgroup members. In reality, experimental subjects often played mixed strategies, indicating that real world signaling is much more fraught with uncertainty than the model indicates.

### Empirical Signaling Strategies with Varying Outgroup Sizes and Costs.

The theory of covert signaling (32, 33) predicts that covert signaling will be more prevalent among 1) individuals in more heterogeneous communities or with minority status and 2) individuals who face higher costs from being recognized as dissimilar. The mathematical model we present above further predicts that in our particular experiment the first effect will be larger than the second one. For our first prediction, we explored conceptualizations of the outgroup as either cross-partisans (e.g., left leaning versus right leaning) or copartisans (e.g., far left versus moderate left).

**Fig. 5.**
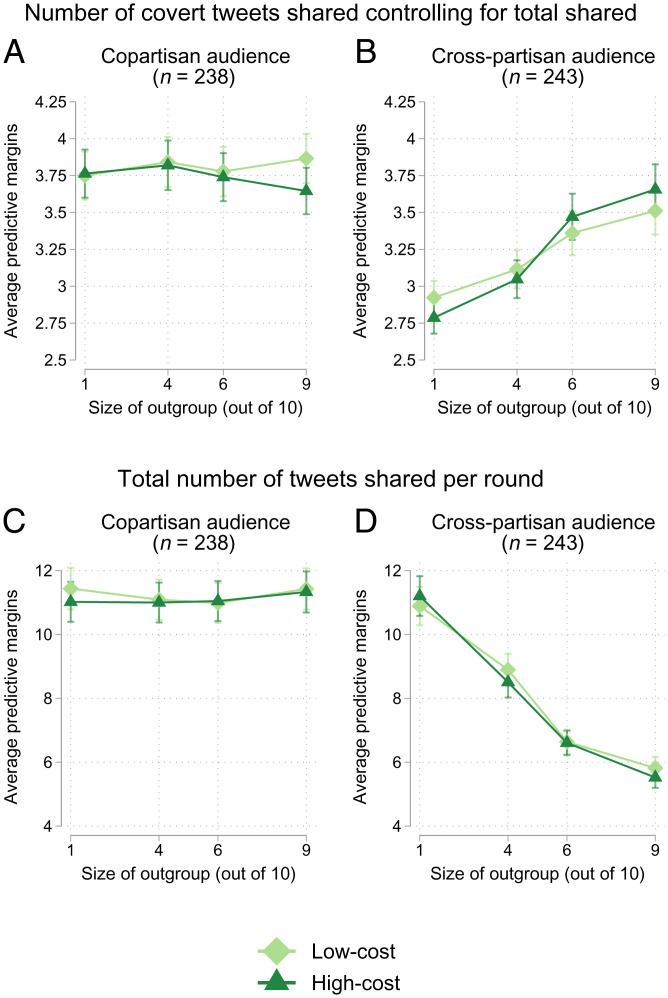
Average predictive margins for the total number of covert (*A* and *B*) and total (*C* and *D*) tweets shared by cost of dislikes across different outgroup sizes, *n* = 481. The outgroup is either composed of cross-partisans (*A* and *C*) or copartisans (*B* and *D*). Estimates from mixed-effects Poisson models, averaging the predicted number of tweets shared across sample values for the number of tweets shared, political leaning, age, gender, race, and education. Error bars represent SEs of the mean.

To formally compare the effect of outgroup size (one or nine audience members out of 10) and cost of dislikes (0.5 or one cent) within individuals and between individuals, we estimated a mixed-effects Poisson model on the count of tweets shared per round, with each outgroup experimental condition nested within individuals and random intercepts for each individual. We used a Poisson distribution to approximate the distribution of the number of covert tweets shared and total number of tweets shared in our sample (*SI Appendix*, Fig.S9). In these models, we controlled for the participants’ age, gender, race, education, and political group (descriptive statistics in *SI Appendix*, TableS8). Fig. 5 presents the average predictive margins for the number of total and covert tweets shared across outgroup sizes and costs. To create these predictive margins, we averaged the predicted number of tweets derived from our full statistical model (*SI Appendix*, section 4.3) across the distribution of gender, age, race, and education in our sample. Our estimates and CIs, therefore, represent the effects for a population similar to our sample: politically engaged and social media–literate Americans across the political spectrum (see *SI Appendix*, section 2 for our selection criteria). The resulting average marginal effect (AME) of outgroup size on the use of covert tweets represents the difference between the average predictive margins between two specific values of outgroup size. We used the *Spost13* package (62) to formally compare these effects.

In line with our first theoretical prediction, we found that participants used more covert signaling as the number of outgroup individuals in the audience increased, as long as the outgroup was conceptualized as cross-partisans (Fig. 5*B*). As the size of the cross-partisan audience increased from one outgroup member to nine, participants tended to use a higher proportion of covert tweets ($AME_{\mathrm{outgroup}}=0.73,95\%CI=[0.47,0.99]$). Even though being the extreme minority was associated with the highest proportion of covert tweets shared, there was also a significant difference between situations with audiences of four outgroup members and six outgroup members in the high-cost condition ($AME_{\mathrm{outgroup}}=0.42,95\%CI=[0.08,0.77]$). This strategy was effective. In *SI Appendix*, we show that participants who changed their signaling strategy, in line with theoretical predictions in the cross-partisan condition, did receive higher bonuses at the end of the experiment (*SI Appendix*, Fig. S8).

There was no effect of outgroup size on the use of covert tweets when the outgroup consisted entirely of members of the same political party, copartisans holding either more moderate or more radical views (Fig. 5*A*, Bayes Factor for null, $BF_{01}=e^{\Delta BIC_{10}/2}=43.15$). In the context of the behavioral experiment, the difference between radicals and moderates within the same party was likely not salient enough to lead to changing strategies across network contexts, and only the cross-partisan condition followed the expectation of the covert signaling identity. This also reflects the fact that the covert and overt tweets used in the experiment were mostly related to cross-partisan disputes. Relatedly, the lack of a copartisan effect may also reflect increased perceptions of cross-partisans as members of an “outgroup” compared with copartisans of varying intensity, particularly during the time the experiment was run.

We did not find strong support for our second theoretical prediction regarding a difference in signaling strategy based on costs. When presented with a higher cost of dislikes in the context of cross-partisan audiences, participants used slightly fewer covert tweets when there was only one outgroup member in the audience. They similarly used slightly more covert tweets compared to lower-cost conditions when there were nine outgroup members in the audience. However, these differences are not significant, and there is, overall, not a strong effect of cost on the relationship between outgroup size and signaling ($BF_{01}=16.83$). The lack of effect likely reflects the small difference in possible payouts between cost conditions—only 20 cents—which may not have been sufficient to induce a strong behavioral change. This is somewhat consistent with the mathematical model of our experiment, which predicted a small effect of cost.

Participants also became more discerning as the size of the outgroup increased. As seen in the cross-partisan condition in Fig. 5*D*, as the size of the outgroup increased from one to nine, participants shared fewer tweets overall ($AME_{\mathrm{outgroup}}=-5.38,95\%CI=[-6.04,-4.72]$). This was true in both the high- and low-cost conditions. However, given the results from Fig. 5*B*, we know that this reduction in the total number of tweets shared was mostly due to reducing the number of overt tweets shared and retaining a few covert tweets. In additional analyses, we explored whether participants’ signaling strategies varied by political literacy, familiarity with Twitter, and demographics (age, gender, race, and education), finding no reliable differences between groups. We do find some subtle differences in signaling behavior across the four political groups studied, as described next.

**Fig. 6.**
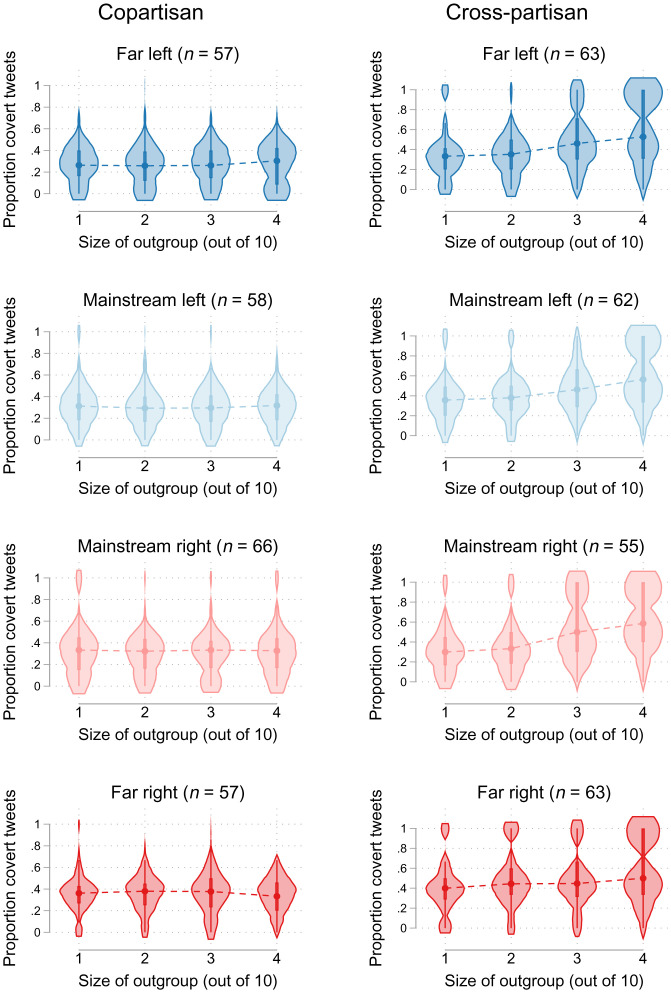
Distribution of the proportion of covert tweets shared in each round across each cost and outgroup size condition, comparing participants who shared tweets to a copartisan audience and cross-partisan audience and separating participants who were determined to be far left, mainstream left, mainstream right, and far right: *n* = 481. Violin plots include a marker for the median of the data, a box for the interquartile range, and spikes extending to the upper- and lower-adjacent values, all overlaid by a density plot using the Epanechnikov kernel function.

The main results hold descriptively across all four political groups (Fig. 6): As the proportion of the audience consisting of outgroup (cross-partisan) individuals increased, participants shared relatively more covert tweets (Fig. 6, *Right*), with no consistent differences observed for purely copartisan audiences in which the outgroup was defined only by the far-left/right mainstream split (Fig. 6, *Left*).

There were small variations across the four political groups. In the copartisan condition, participants on the right side of the political spectrum used slightly more covert tweets compared to participants on the left. In the cross-partisan condition, the patterns also varied between moderates and radicals. On the one hand, mainstream right and left individuals had quite an increase in their covert signaling when the number of outgroup audience members changed from four to six. On the other hand, radical right and left participants exhibited a more linear increase in their proportion of shared covert tweets by outgroup size. Radical right and left participants were also more bimodal, with one subgroup sharing almost only covert tweets when they were in the minority and another subgroup sharing a relatively lower proportion of covert tweets. The difference between moderates and more extreme participants was greater on the right compared to the left. Far-right participants had fewer changes in their signaling strategy over varying outgroup sizes compared to moderate right participants. Though we could not determine an explanation for these differences with such small groups, our results point to some variation in the perceived ability to assort with similar others and the perceived cost of signaling.

## Discussion

We have shown how individuals may be strategically altering their online communication based on their likely audiences and that they do so in ways mostly consistent with the theory of covert signaling. This work connects to older theories of both identity signaling (2, 61, 63) and audience design (37, 38, 64, 65) but extends these theories by situating signaling behaviors in a sociocultural milieu and making clearer predictions based on context. The theory of covert signaling is inherently challenging to test empirically, because covert signals are, by definition, not easily perceived by outsiders who lack “insider” knowledge. Our methods were designed to overcome this difficulty by relying on differences in the responses among raters with different identity affiliations.

The theory predicts that individuals should use more covert signaling in more heterogeneous groups or when they are in the minority. We found support for this prediction in the ways people shared political speech in a behavioral experiment. We observed the highest levels of covert signaling when audiences consisted almost entirely of cross-partisans, supporting the notion that covert signaling is a strategy for avoiding detection by hostile outgroup members. Of note, we selected tweets for our study at a time of heightened partisan divisions: the four weeks preceding the 2020 US presidential election. Consequently, these tweets mostly discussed the opposing political party. This focus was reflected in our behavioral experiment, in which we did not observe an effect of audience composition when all members were (more or less extreme) copartisans. In that societal context, participants might have perceived the cost of dislikes to be minimal and have likely focused on partisan disputes in their real-life conversations happening around that time. Future work testing the theory of covert signaling should also examine signaling strategies in copartisan conversations during times of salient intragroup political divisions.

We found no significant effect of cost in our experiment. In previously published, formal models (32, 33), costs translated into observable differences in payoffs of agents, which affected their likelihood of becoming a target for imitation by others. It is likely that the differences in costs in our experimental conditions—which involved a maximum difference in losses of only 20 cents between high- and low-cost conditions—were insufficient to motivate strong differences in behavior. The mathematical model of our experimental design, described in *Results*, helps to explain why the effect of audience composition (i.e., how much of the audience are outgroup members) should be so much stronger than the effect of cost. However, the effect of cost is even smaller than we expected. Future work should take into account stronger differences in potential losses to investigate differences in the cost of dislikes.

We found small differences in the use of covert tweets between far-left/right participants and moderates. The theoretical models that inspired this study (32, 33) make no explicit predictions about differences between political groups, though differences could be explained from the perceived ability to assort with similar others or in the perceived cost of being exposed to dissimilar others. In this case, some of the more politically extreme participants might consider the cost of being dissimilar to be high in the cross-partisan condition no matter the size of the outgroup, as they might consider other left- or right-leaning people as an outgroup as well. This might be even more true for far-right participants who tend to consider Twitter as a left-leaning community. We hope to continue investigating more subtle differences in perceived outgroup members and related costs.

Our study reflects the complexity of how strategies of identity signaling can vary across communities and time. In the real world, including Twitter, communities are highly assortative, and individuals can often restrict interaction partners to members of their ingroups (66–68). Twitter networks, therefore, tend to be segregated, with conversations mostly occurring within coarse-grained political groups. This assortment drove our methods for the selection of tweets reflecting political identity. Future work should explore how changes in assortativity in and out of social media influences the use of covert signaling online.

Another direction for future empirical work on covert signaling in more natural settings is the analysis of the dynamic relationship between covert signaling and social networks. Specifically, individuals who are more likely to use overt signaling might end up in more homogeneous networks, either by choice or because they are shunned by disagreeing audiences. In our experimental design, the audience composition was fixed, but in reality, networks change after individuals express their opinions and identities over time. Signal type and utility can also be affected by changes in the network. For example, as familiarity with initially covert signals grows beyond insider networks, those signals can become overt [as with the recent case of the anti-Biden catchphrase “Let’s go Brandon.” (69)]. In our study, participants did not receive direct feedback after each round so did not have the chance to refine their signaling over time, but we hope to continue to refine our experiment to investigate further the coevolutionary dynamics of signals and networks.

Our empirical study could not discern intent on the part of either signalers or receivers. Signalers did change their signaling strategy by using proportionally more covert tweets as the audience contained more outgroup members. We tried to construct a situation that would resemble identity signaling, in which each participant could only share tweets from their own political side and were rewarded for being only correctly perceived by outgroup members. However, the participants may have been trying to maximize payoffs irrespective of sharing their identity. To further test the theory of covert signaling, we should study situations in which we know individuals want to share their identity. Furthermore, we should differentiate outgroup receivers who are “churlish,” disliking any signal that they do not see as ingroup (33), from those who actually understand the true intent of a covert tweet. We hope to continue disentangling the intents of both identity signalers and receivers, and develop a theory that takes into account these nuances.

This work provides three specific pathways to develop and refine the theory of covert signaling. First, our findings shed light on the complexities of measuring the covertness of signals in a real-world setting. Our study design assumed a relatively clear dichotomy between overt and covert signaling, in line with prior modeling work (32, 33). However, our results indicate that the covert–overt distinction is likely more continuous than dichotomous and that signal vocabularies may be heterogeneously distributed even among ingroup members. For example, even though outgroup members mostly disliked overt tweets, some disliked covert tweets as well. In that case, the utility of signaling with a given covertness level will depend on the cost–benefit tradeoffs regarding successful ingroup detection versus the costs of outgroup detection. The continuous nature of covertness is an important topic for future theoretical and empirical work.

Second, our findings highlight other strategies that individuals might use when signaling in heterogeneous networks beyond switching from overt to covert signals. We find here that participants were overall more cautious with what they shared in heterogeneous environments or when in the minority, sharing fewer tweets overall. In case of a potential higher cost of being disliked by the outgroup, many might believe that the benefits of identity signaling to the ingroup might not be strong enough to compensate for the potential cost of being disliked by the outgroup and thus decide to reduce the amount of signaling altogether. Future iterations of the theory of covert signaling could model not only the benefits of covert versus overt signals but also consider the decision to signal in the first place.

Finally, in large, multicultural societies, identity is both critical and complicated. It is multidimensional and contextual (3, 70), and even political identity is more nuanced than the simple unidimensional left–right consideration implicit in this work. Indeed, our experiment did not afford participants the oblique choice to signal about something related to a different identity. The multidimensional nature of identity is discussed at length in a previous theory paper on covert signaling (3) but not included in the models for tractability. Instead of using fewer tweets, our participants could have preferred to share different types of tweets. Future modeling and empirical testing of the theory of covert signaling should investigate how having multiple dimensions for identity signaling can influence the decision to use covert or overt signals.

Our analysis suggests that people adjust their political speech according to strategic incentives, in ways predicted by our formal mathematical and computational models. Our work also points to specific areas in which the theory of covert signaling could be improved. We believe this work provides support for calls to increase the integration of formal modeling with mainstream social science research (71–76). Formal models provide guidelines for assessing the scope of a theory—the constraints required for the theory to apply (58). Our study was designed explicitly to test predictions from a formal model of covert signaling. To enable further theory development, additional models of overt and covert political signaling could be formally developed and their predictions compared with those of the current model.

Our work highlights that even deciding whether to classify communication as “political” is inherently context dependent. The significance of a statement depends on the circumstances in which it is communicated and on the background knowledge of the receiver. This indicates that automated methods to detect political speech online (e.g., ref. 77) are likely restricted to detecting only overt speech. Such classifiers will likely miss most if not all covert signals and will therefore ignore the strategic use of information that is simultaneously interpreted in different ways by different audience members. When speech is public, audiences are diverse, and identity matters, at least some identity-related speech is likely to be encrypted.

Overt political speech at its worst amounts to hate speech, in which cross-partisans are vilified and even dehumanized. At minimum, public political speech in the United States increasingly reflects affective polarization, in which individuals draw clear culture differences between “us” and “them” along partisan lines (5, 23, 78) and may share information for the purpose of declaring coalitional alignment rather than to communicate knowledge of the world (7, 79). The consequences of this sort of continued polarization are probably stark. Our study suggests that overtly parochial speech will be more common when individuals can more easily assort into networks of like-minded individuals, particularly when those individuals are politically engaged with partisan media. There is evidence that, at least in the United States, media is growing not only increasingly partisan but is actively stoking the fires of partisanship (6, 80).

For a diverse society to function effectively and cooperatively despite cultural differences, people must maintain some level of civility in public discourse, in which members of other groups are respected even if they are not always included. We should accordingly expect coded language and other covert identity signaling to be common, because diversity implies a variety of norms, goals, and experiences among individuals who will at least sometimes seek similar company. Covert signaling may be a sign of a functioning cosmopolitanism (33). However, it is also possible that covert signaling is indicative that some people have reasons to fear having their true identities publicly revealed. The benefit of covert signaling therefore may depend on the domains in which people interact and cooperate and the topics that are allowed or proscribed in public discourse. Studies of covert signaling are increasingly important to understand the contextual implications of communication in a culturally diverse landscape.

## Materials and Methods

To test our theoretical predictions, we designed an empirical pipeline that consisted of 1) selecting Twitter users who might be more likely to use covert or overt political speech and downloading their tweets, 2) rating a selection of tweets on different dimensions and marking a subset as more likely to be covert or overt, and 3) conducting a behavioral experiment with the selected covert and over tweets to test whether people indeed use them to communicate their political beliefs in theoretically predicted conditions.

Our study took place between September 15 and November 1, 2020. Our study was deemed exempt by University of California Merced’s Institutional Review Board office (UCM2020-65) on May 5, 2020. The anonymized data and code are available on the Open Science Framework (OSF): DOI 10.17605/OSF.IO/XMZRA (81).

### Twitter Data Collection.

We developed a process for selecting candidate tweets that would maximize the proportion of overt and covert political signals on both sides of the political spectrum (Fig. 2*A*). To download these tweets, we also chose a time frame that had intensified political discussions online: September 15 through October 12, 2020. Overall, our selection process was designed to both minimize the amount of work needed to rate all the tweets downloaded and maximize the potential number of covert and overt tweets to use in the behavioral experiment.

First, we selected seed Twitter accounts from a list of followers of eight news accounts representing far-left and far-right factions of the US political landscape on Twitter (*SI Appendix,* Table S1). These news accounts generally corresponded to the progressive left wing, more closely affiliated with the Democratic party, and the Trumpist or Tea Party right wing, more closely affiliated with the Republican Party. As Twitter’s application programming interface (API) limits the download of lists of followers, we randomly sampled 10,000 followers of each of the eight news accounts. Any account that had not tweeted 4 wk prior to sampling was rejected and replaced through continued sampling. We were able to download the list of followers for 73,869 of these accounts (fewer than the 80,000 originally planned due to technical limitations).

Second, we developed a schema for classifying the political orientation of each seed account based on their media diet as represented by the proportion of far-left or -right political news sources they followed. We identified highly “polarized” US-based news outlets using data from mediabiasfactcheck.com and selected those whose Twitter accounts had at least 10,000 followers. This produced a set of 43 far-left news accounts and 50 far-right news accounts (*SI Appendix,* Table S1). While these sets of accounts might not represent a coherent political identity, they can still be combined to approximate the orientation and degree of political engagement of a single Twitter user. We downloaded the follower list for each of the news accounts and ranked far-left/right seed accounts by the proportion of far-left/right news accounts they followed from our list. We selected the top 20% of these seed accounts on the left and right, representing the most “engaged” accounts and thus more likely to use political identity signaling for a total of 16,398 engaged users.

Third, we classified the networks of each of the engaged seed accounts as either politically homogeneous or heterogeneous. We considered different operationalizations of political heterogeneity. One potential operationalization of heterogeneity is the relative proportion of cross-partisans in one’s follower network. However, we found that cross-partisan follower relationships (as identified by media diet) were rare, and embeddedness in a cross-partisan follower network was practically nonexistent. Instead, we operationalize homogeneity and heterogeneity in terms of the extent of engagement with copartisan far-left/right news accounts. Homogeneous follower networks are those that are about as engaged or committed to strong partisanship as their seed accounts, while heterogeneous follower networks are much less engaged than their seed accounts. Drawing on the theory of covert signaling, we expect seed accounts with heterogeneous networks to be less likely to overtly share their political identity than seed accounts with homogeneous networks.

Followers of our seed accounts were labeled as engaged if they followed at least as many news accounts as the 50th percentile of our initial pool of seed accounts (three for the left and four for the right) and as disengaged if they followed at most one far-left/right news site. In order to have a pool of tweets with both many overt and covert tweets, we selected seed accounts that had the most homogeneous and heterogeneous networks. Specifically, we selected the top 20% of engaged seed accounts, ranked by the proportion of engaged followers in their networks, and labeled them as having “homogeneous networks” (as they were politically engaged themselves and had a high proportion of engaged followers). Then, we selected the top 20% of engaged seed accounts, ranked by the proportion of disengaged followers in their networks, and labeled them as having “heterogeneous networks” (as they were politically engaged themselves and had a high proportion of disengaged followers). We ended up with 1,834 far-left accounts and 1,446 far-right engaged accounts with either homogeneous or heterogeneous follower networks.

Fourth, we downloaded tweets from the resulting four groups of engaged seed accounts (far left/right with homogeneous/heterogeneous follower networks). Using the Twitter API, we downloaded up to the maximum number of available tweets from each user (3,200) in the 4 wk leading up to the behavioral experiment. We filtered out tweets that would be difficult to understand for independent raters (see *Rating and Classification of Tweets*), removing tweets that were posted more than 6 wk prior, were replies to other users, contained images or news article links, were not in English, or were too short (fewer than 5 words or 50 characters, unless they contained hashtags which tend to be high-content, contextualizing signals). To further improve legibility, we deleted all other links from tweets. Finally, we removed retweets to avoid duplicates. This procedure produced between 1,303 and 2,100 tweets for each of the four groups of seed accounts (far-left/right with homogeneous/heterogeneous follower networks) for a total of 6,594 tweets. Of those, we randomly selected 1,303 or 1,304 tweets for each group for a total of 5,215 tweets (originating from 1,409 seed accounts) to be rated and classified as covert or overt, as described in the following sections.

### Rating and Classification of Tweets.

To determine whether tweets downloaded were overt or covert, we recruited human raters (Fig. 2*B*). To establish a pool of raters, we preselected individuals from Mechanical Turk whose political orientation was either far left, moderate left, moderate right, or far right and who had at least a minimum amount of political literacy. We determined their political leaning based on news diet, self-reported political identification, and views on radical political movements. Political literacy was determined using self-reports of frequency of following the news, a political quiz, and familiarity with news discussions on social media. We describe the participant selection process in detail in *SI Appendix*, section 2. We invited from this preselected pool a sample of 2,695 raters. Of those, 1,992 raters responded (*n* = 483 far left, 533 moderate left, 461 far right, and 515 moderate right).

We asked raters several questions about each tweet to determine the tweet’s position on two dimensions: political orientation and affective response (see Questionnaire in *SI Appendix*, section 3). For the political orientation dimension, raters were asked to estimate the perceived political orientation of the tweets’ authors on a seven-point scale from extreme left to extreme right or to answer “not political.” For the affective response dimension, raters were asked two questions: to estimate how negatively they felt toward a tweet and how offensive the tweet would be to people similar to themselves (from very offensive to not offensive). The two ratings were highly correlated (*r* =.70) and were averaged in one summative scale. We pretested a third question on the offensiveness for other groups but excluded it, given that it did not clearly reflect how the ingroup or outgroup reacted to the political signal.

Each rater received 50 randomly selected tweets, a number that, according to our pretests, one person could evaluate in 20 min, approximating the feasible duration for an online study (82). Of the 5,215 tweets selected, as described in the previous step, we obtained ratings from at least three raters from each of the four political leaning groups (far left, moderate left, moderate right, and far right) for 4,752 tweets. Each tweet was given four scores for the two dimensions, reflecting the average response of the four groups of raters (*SI Appendix*, section 3). To investigate interrater reliability, we fitted a random effects linear model to probe how much variation in the political leaning ratings could be attributed to individual raters. The multilevel regression model estimates random effects for tweet, rater, and partisanship of rater (our four groups). We used Shapley regression to decompose the explained variance (in this case, McFadden’s pseudo-R^2^). The multilevel regression accounts for 50.0% of the total variance. Of this, 89.3% of the variance can be attributed to the tweets themselves. Raters account for 10.3% of the explained variance, which is just 5.2% of the total variance. Thus, we conclude that while each rater saw a different subset of tweets and rating is somewhat noisy, interrater reliability was high.

We used a set of criteria (*SI Appendix,* Table S6) drawing on the theoretical framework of Smaldino and colleagues (32, 33) to use these scores to select covert and overt tweets. Under the assumption that partisans with extreme views were likely to be highly engaged and therefore more likely to be attuned to covert copartisan political signals, we expected that extreme partisans would recognize covert signals as supporting a particular political side and would experience a strong affective response, while more moderate partisans would show no consistent response on these dimensions. In contrast, overt tweets should be recognized by most people as advocating a particular political side and should be liked if they supported one’s own political identity. Note that our assumption that extreme partisans are more likely to be attuned to covert copartisan political signals deviates from standard conceptions of ingroup and outgroup signaling. However, in a highly politicized environment such as the contemporary United States, extreme partisans are often motivated primarily by their opposition to the outgroup (7, 23, 78) and are therefore highly attuned to signals of outgroup membership and unlikely to be fooled by covert signals. In contrast, moderate partisans are less likely to have detailed insider knowledge of the ingroup signals used by cross-partisans.

Accordingly, we marked tweets as potentially covert if they satisfied the following five theory-based criteria (Fig. 2*B* and *SI Appendix,* Table S6): large copartisan difference in tweets’ perceived political orientation and affective scores, neutral ratings of political orientation given by moderates, neutral or positive affective score by moderates, and political content recognized by far copartisan raters. We marked tweets as potentially overt if they satisfied the following four theory-based criteria (see *SI Appendix,* Table S6 for details): small cross-partisan difference in tweets’ political orientation score, large difference in cross-partisan and small difference in copartisan affective score, and political content recognized by all raters. We set up percentile cutoffs to obtain around 30 tweets that had the highest probability to represent each of the four categories—covert left (37), covert right (34), overt left (56), and overt right tweets (37)—for a total of 164 automatically preselected tweets. From these, we selected 20 tweets (the number that was feasible timewise for our behavioral experiment) in each of the four categories using independent evaluations of overall covertness/overtness by each of the four authors, followed by a joint discussion. Two right-leaning tweets were erroneously included as covert tweets for the behavioral experiment and were subsequently recoded as neither covert nor overt for the analysis.

In *SI Appendix*, section 3.1, we show that the average group-level response for covert and overt tweets across dimensions match our criteria. Furthermore, we examine in *SI Appendix*, section 3.2 whether covert and overt tweets appear in more homogeneous or heterogeneous Twitter networks and find supportive results (i.e., a greater fraction of political tweets from users with heterogeneous networks were covert than was true for homogeneous networks) (*SI Appendix*, Fig. S5). Together, these show our ratings and criteria carry useful information related to political signaling and suggest that covert and overt tweets should be perceived differently by participants in our behavioral experiment. While our methods were designed to select the most likely covert and overt tweets in our sample, we refer to these tweets as “covert” and “overt” in our results for simplicity.

### Behavioral Experiment.

We used the tweets selected as overt and covert political signals in the previous section to conduct an experimental study testing our theoretical predictions in a controlled setting (Fig. 2*C*). We designed a behavioral experiment to test two predictions derived from the theory of covert signaling: 1) Individuals should use more covert signaling when in more heterogeneous groups or when they are in the minority, and 2) individuals should use more covert signaling when the costs of being recognized as dissimilar are higher.

We selected a subset of 240 participants from the pool of raters described in *SI Appendix*, section 2 who were screened as being far-left/right in their political views and 240 who were more moderate, with an equal number of participants with left and right political identities in far and moderate groups. In the first part of the experiment, participants were asked if they liked, disliked, or were neutral toward each of the 80 left- and right-leaning tweets selected for this stage. In the second part of the experiment, participants were asked to select and share tweets to an audience of 10 other participants. Each participant saw a randomly sorted list of 40 only left- or right-leaning tweets, matching their own political orientation, split between covert and overt messages. Participants were paid a baseline of $3.40 and were given a bonus for each audience member who liked the majority of the tweets they shared and charged a cost for each audience member who disliked the majority of their tweets. The instructions to the participants are shown in *SI Appendix*, Figs. S6 and S7.

Participants were randomly split in two experimental groups: one having a strictly copartisan audience (in which audience members were identified as either extreme or moderate copartisans) and one having a partly cross-partisan audience (in which audience members were identified simply as either co- or cross-partisans). Each group participated in eight successive rounds, randomly ordered to vary two within-subject factors: 1) the number of people in the audience with political leanings different from their own (one, four, six, or nine people in the outgroup, either more radical or mainstream for participants with a copartisan audience or from the opposing political side for participants with a cross-partisan audience) and 2) the cost of dislikes, either a low-cost condition in which the cost of dislikes (0.5 cents) was half of the bonus for likes (1 cent) or a high-cost condition in which the cost of dislikes was the same as the bonus for likes (both 1 cent). Bonuses were calculated based on the actual likes and dislikes recorded from participants in the first part of the experiment.

## Supplementary Material

Supplementary File

## Data Availability

Anonymized tweet- and individual-level data in csv or dta formats, alongside the code for all analyses, have been deposited on a publicly accessible database on OSF (10.17605/OSF.IO/XMZRA) (81). In order to respect the anonymity of Twitter users, we do not share their user IDs and tweet texts.
