# Supplementary material for: Strategic identity signaling in heterogeneous networks
Source: Proc Natl Acad Sci U S A. 2022 Mar 3;119(10):e2117898119. doi: 10.1073/pnas.2117898119 (PMC8915961; doi:10.1073/pnas.2117898119)
Supplement: Supplementary File [file pnas.2117898119.sapp.pdf]

SI Appendix for "Strategic Identity Signaling in Heterogeneous Networks"

1 Twitter data collection

Table S1: News sites used to determine Twitter account engagement with far-left/right politics. Initial 8 news accounts (from whom followers were drawn to provide the initial 80,000 seed accounts) are in bold.

Left Sites	Twitter Handle	# Followers
Salon	@Salon	975,000
Human Rights Campaign	@HRC	823,100
Think Progress	@thinkprogress	808,500
**Democracy Now**	@democracynow	742,000
Splinter	@splinter_news	544,800
**Young Turks**	@TheYoungTurks	414,000
Jezebel	@Jezebel	318,600
Jacobin	@jacobinmag	298,100
Daily Kos	@dailykos	289,300
Feministing	@feministing	230,200
Raw Story	@RawStory	216,600
Right Wing Watch	@RightWingWatch	178,800
TruthOut	@truthout	178,200
New Republic	@newrepublic	172,100
**Chapo Trap House**	@CHAPOTRAPHOUSE	146,000
Alternet	@AlterNet	139,100
Electronic Intifada	@intifada	115,300
**Truth Dig**	@Truthdig	96,000
Code Pink	@codepink	95,400
Crooks and Liars	@crooksandliars	93,400
It's Going Down	@IGD_News	87,700
Towleroad	@tlrd	77,300
Common Dreams	@commondreams	76,300
Dissent Magazine	@DissentMag	70,100
CounterPunch	@NatCounterPunch	69,200
Free Press	@freepress	58,900

Left Sites	Twitter Handle	# Followers
Libcom.org	@libcomorg	52,400
Care2	@Care2	51,900
In These Times	@inthesetimesmag	43,000
American Bridge 21st Century	@American_Bridge	38,100
Cop Block	@CopBlock	26,200
Institute for Policy Studies	@IPS_DC	18,500
In Defence of Marxism	@marxistcom	16,200
Earth First Journal	@efjournal	15,600
Democracy Chronicles	@demchron	14,100
North99	@wearenorth99	14,000
Workers World	@workersworld	13,100
Act.TV	@actdottv	12,800
Peacock Panache	@PeacockPanache	12,200
Occupy.com	@occupy	11,200
Egberto Willies	@EgbertoWillies	10,500
Center for a Stateless Society	@c4ssdotorg	10,100
Democratic Underground	@demunderground	10,000

Right Sites	Twitter Handle	# Followers
**Breitbart**	@BreitbartNews	1,300,000
**The Blaze**	@theblaze	725,000
Daily Caller	@DailyCaller	719,600
Heritage Foundation	@Heritage	670,200
**Daily Wire**	@realDailyWire	416,000
National Review	@NRO	347,000
FreedomWorks	@FreedomWorks	291,100
The Federalist	@FDRLST	262,000
Life News	@LifeNewsHQ	245,400
**Red State**	@RedState	229,000
Conservative Review	@CR	199,300
The Rebel News	@RebelNewsOnline	186,100
Christian Post	@ChristianPost	172,000
Newsmax	@newsmax	155,700
Washington Free Beacon	@FreeBeacon	120,400
Independent Journal Review	@TheIJR	115,100
Chicks on the Right	@chicksonright	98,900
Daily Signal	@DailySignal	71,400
Far Left Watch	@FarLeftWatch	60,900
Federalist Society	@FedSoc	60,100
Human Events	@HumanEvents	51,600
Law Enforcement Today	@LawEnforceToday	48,100
BizPac Review	@BIZPACReview	46,700

Right Sites	Twitter Handle	# Followers
American Spectator	@amspectator	46,100
Christian Coalition of America	@ccoalition	42,400
The Right Scoop	@trscoop	41,000
Numbers USA	@NumbersUSA	36,800
Lifezette	@LifeZette	34,300
Energy Citizens	@energycitizens	33,700
Accuracy in Media	@AccuracyInMedia	30,100
Politichicks	@ThePolitiChicks	25,100
Tenth Amendment Center	@TenthAmendment	23,000
The Resurgent	@resurgent	21,800
Richochet	@Ricochet	21,100
American Greatness	@theamgreatness	20,700
Ami Magazine	@Ami_Magazine	20,500
Bearing Arms	@BearingArmsCom	19,100
Weasel Zippers	@weaselzippers	18,500
Claremont Institute	@ClaremontInst	17,700
Crisis Magazine	@CrisisMag	16,600
The College Fix	@CollegeFix	16,300
PanAm Post	@PanAmPost	15,500
Disrn	@DisrnNews	14,800
Liberty Nation	@libertynation	13,700
Media Equalizer	@MediaEqualizer	12,800
Communities Digital News	@CommDigiNews	12,400
The American Mind	@theammind	12,000
Intellectual Takeout	@intellectualTO	11,600
Media Trackers	@mediatrackers	11,100
Citizen Free Press	@CitizenFreePres	10,200

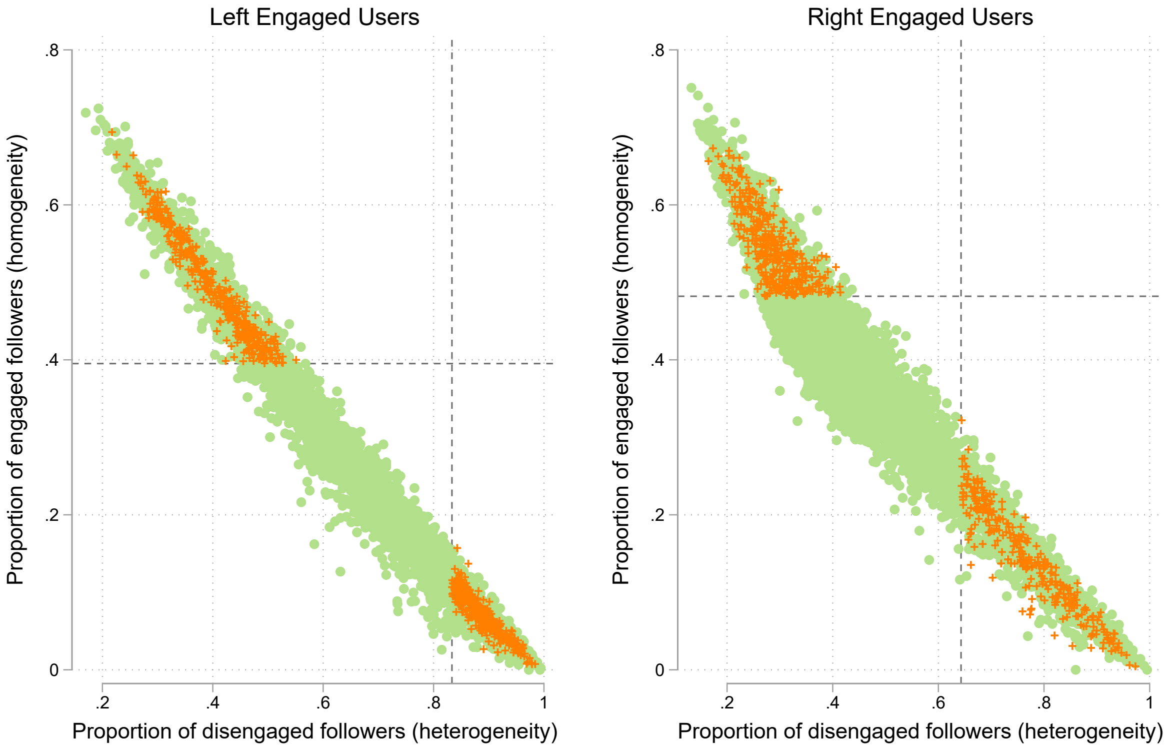

Figure S1: Relationship between heterogeneity and homogeneity scores among the initially considered 16,398 Twitter users (green dots; $r = .99$). Orange crosses denote 1,409 Twitter users that were selected for the rating study because they were in the top 20% by either homogeneity or heterogeneity scores and had tweets that matched our selection criterion.

2 Participant selection

We used CloudResearch, a panel service associated with Mechanical Turk (MTurk), to select 3,027 individuals who were identified in CloudResearch screening survey as having a "moderate", "very liberal", or "very conservative" political orientation. They were invited to a brief survey in which they were asked to solve a political literacy quiz and report on their news-following habits, the political groups they identify with, their familiarity with different political groups and movements, and their opinions about different political issues.

Based on the survey responses, we selected four groups of raters as described in Figure S2. To select far-left and far-right raters, we focused on their preferred news sources and identities. We included individuals who reported using at least one far-left or far-right source regularly to keep up with the news (see TableS3 in the *SI Appendix*) or who identified with a far-left or far-right identity (see Table S4 in the *SI Appendix*). In addition, we screened out participants who identified with inconsistent political views (e.g., followed a far-right news source but self-described as very liberal) or with inconsistent political identities (e.g., identified with a far-left identity and as a Republican). Although it may be perfectly reasonable to follow cross-partisan news accounts, we excluded these individuals to reduce a potential source of noise in our data.

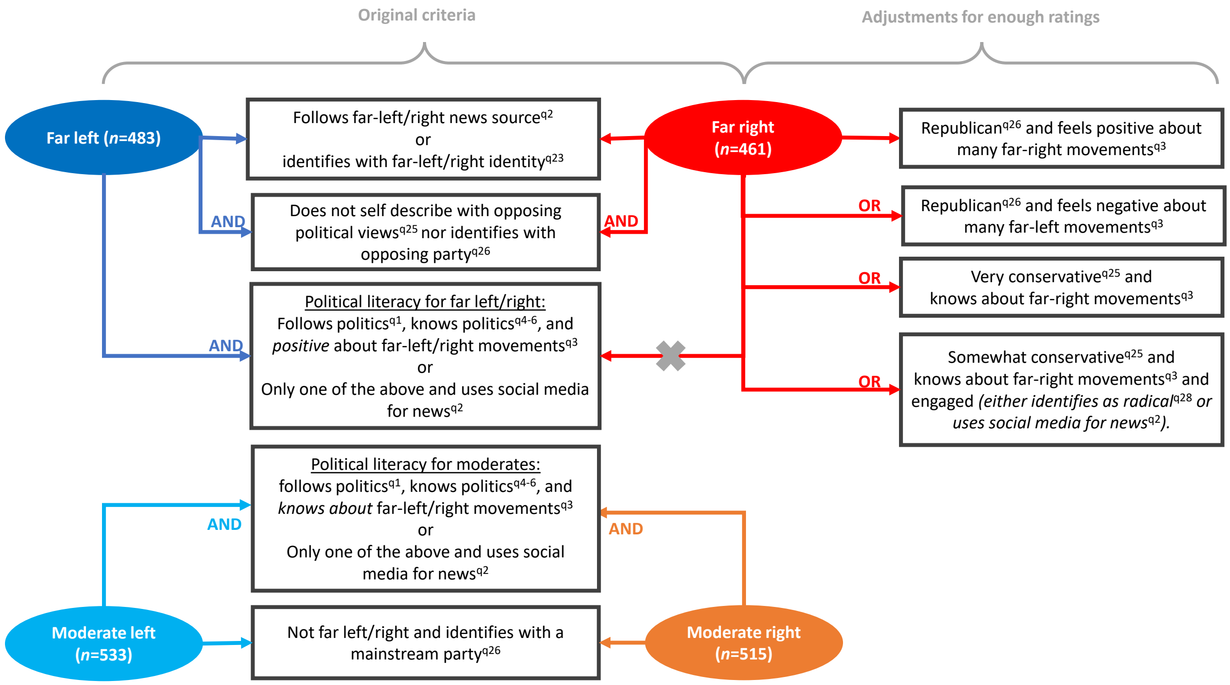

Figure S2: The process of selection of raters. Superscripts refer to the survey question (see Questionnaire below) used for each criterion.

In order to select raters with at least a minimum of political literacy, we selected participants who reported following the news at least once a week, answered two out of three questions correctly in the political literacy quiz, and were in the top 50% by their positivity toward their own far-left/right political movements (see Table S5 in the *SI Appendix*) . Because our analysis focused on rating or sharing tweets, we wanted to oversample people

familiar with social media and therefore we also added to the pool participants who had only one of the criteria of political literacy but listed a social media site as their regular source of news (Twitter, Facebook, Reddit, 4chan, 8kun, ctree, and forums). To select moderate left and right raters, we included all participants who identified as Democrat or Republican and were not marked as having a far-left/right ideology. In addition, we used the same political literacy criteria as for far-left/right participants, except we selected those who were in the top 75% in knowledge far-left/right movements (regardless of feeling positive about them).

Given these criteria, our pool of potential raters included fewer far-right participants compared to all other groups. This is not surprising given that people who participate in Amazon's Mechanical Turk platform tend to lean Democrat [2]. We therefore broadened our criteria for far-right raters, while still retaining the focus on far-right ideology, identity, and a minimum of political literacy. We included participants who identified as Republican and, compared to all right-leaning participants, were in the top 10% for feeling positive about many far-right movements or top 10% for feeling negative about far-left movements. We also added participants who self-described as very conservative and were in the top 75% of right-leaning individuals according to their knowledge of far-right movements. Finally, we added to the pool participants who identified as somewhat conservative, were in the top 75% according to their knowledge of far-right movements, and were engaged online (either self-identified as radical or used social media for news). We used this pre-selected pool for both the rating of tweets and the behavioral experiment.

Questionnaire

1 Political literacy

[q1] Would you say you follow what's going on in government and public affairs...

- ◯ More than once a day
- ◯ Every day
- ◯ More than once a week
- ◯ Once a week
- ◯ At least once a month
- ◯ Less than once a month
- ◯ Never

2 Political sources

[q2] Which of the following sources do you regularly seek out to keep up with politics and election news?

Select all that apply.

☐ CNN ☐ MSNBC ☐ Fox News ☐ Local news on TV ☐ The Wall Street Journal ☐ The Economist ☐ The New York Times ☐ Washington Post
☐ Local newspaper ☐ Talk radio ☐ NPR ☐ Democracy Now ☐ Jacobin ☐ Breitbart ☐ Current Affairs ☐ National Review
☐ InfoWars ☐ Vox ☐ The Daily Caller ☐ The Nation ☐ Twitter ☐ Facebook
☐ Other (specify) ____

3 Feelings political movements

[q3] Select how you feel about each of the following groups or movements:

	Positive	Neutral	Negative	Never heard of it
American Party of Labor	◯	◯	◯	◯
Anonymous	◯	◯	◯	◯
Black Lives Matters	◯	◯	◯	◯
Democratic Socialists of America	◯	◯	◯	◯
Fraternal Order of Alt-Knights	◯	◯	◯	◯
League of the South	◯	◯	◯	◯

	Positive	Neutral	Negative	Never heard of it
Occupy Wall Street	◯	◯	◯	◯
Unite the Right	◯	◯	◯	◯
Vanguard America	◯	◯	◯	◯
QAnon	◯	◯	◯	◯
Loyal White Knights	◯	◯	◯	◯
Antifa	◯	◯	◯	◯
Workers World Party	◯	◯	◯	◯
American Identitarian	◯	◯	◯	◯
Indivisible	◯	◯	◯	◯
Communist Party USA	◯	◯	◯	◯
Identity Evropa	◯	◯	◯	◯
Stormer Book Club	◯	◯	◯	◯
Traditionalist Worker Party	◯	◯	◯	◯

4 Political questionnaire

In the next three questions, we are looking for your intuitive answers. Please do not look up the correct answers online - ***your answers will have absolutely no***

[q4] To your knowledge, when will the next US presidential elections be held?

- ○ November 2020
- ○ December 2020
- ○ January 2022
- ○ May 2022
- ○ September 2022
- ○ Don't know

[q5] To your knowledge, which party has control of the US House of Representatives?

- ○ Democratic party
- ○ Republican party
- ○ Other
- ○ Don't know

[q6] To your knowledge, which party has control of the US Senate?

- ○ Democratic party
- ○ Republican party
- ○ Other
- ○ Don't know

5 General opinions

The following pairs of statements will help us understand how you feel about a number of issues. For each pair, please tell us which statement comes closer to you

[q7] Which of the following statements comes closest to your view?

○ The government should do more to help needy Americans, even if it means going deeper into debt
vs.
○ The government today can't afford to do much more to help the needy

[q8] Which of the following statements comes closest to your view?

○ Racial discrimination is the main reason why many black people can't get ahead these days
vs.
○ Black people who can't get ahead in this country are mostly responsible for their own condition

[q9] Which of the following statements comes closest to your view?

○ The best way to ensure peace is through military strength
vs.
○ Good diplomacy is the best way to ensure peace

[q10] Which of the following statements comes closest to your view?

○ Government is almost always wasteful and inefficient
vs.
○ Government often does a better job than people give it credit for

[q11] Which of the following statements comes closest to your view?

○ Government regulation of business is necessary to protect the public interest
vs.
○ Government regulation of business usually does more harm than good

[q12] Which of the following statements comes closest to your view?

○ Poor people today have it easy because they can get government benefits without doing anything in return
vs.
○ Poor people have hard lives because government benefits don't go far enough to help them live decently

[q13] Which of the following statements comes closest to your view?

○ Immigrants today strengthen our country because of their hard work and talents
vs.
○ Immigrants today are a burden on our country because they take our jobs, housing and health care

[q14] **Which of the following statements comes closest to your view?**

- ○ Most people who want to get ahead can make it if they're willing to work hard

vs.

- ○ Hard work and determination are no guarantee of success for most people

[q15] **Which of the following statements comes closest to your view?**

- ○ Business corporations make too much profit

vs.

- ○ Most corporations make a fair and reasonable amount of profit

[q16] **Which of the following statements comes closest to your view?**

- ○ Stricter environmental laws and regulations cost too many jobs and hurt the economy

vs.

- ○ Stricter environmental laws and regulations are worth the cost

[q17] **Which of the following statements comes closest to your view?**

- ○ Homosexuality should be accepted by society

vs.

- ○ Homosexuality should be discouraged by society

[q18] **Which of the following statements comes closest to your view?**

- ○ In foreign policy, the U.S. should take into account the interests of its allies even if it means making compromises with them

vs.

- ○ In foreign policy, the U.S. should follow its own national interests even when its allies strongly disagree

[q19] **Which of the following statements comes closest to your view?**

- ○ It's best for the future of our country to be active in world affairs

vs.

- ○ We should pay less attention to problems overseas and concentrate on problems here at home

[q20] **Which of the following statements comes closest to your view?**

- ○ Our country has made the changes needed to give blacks equal rights with whites

vs.

- ○ Our country needs to continue making changes to give blacks equal rights with whites

[q21] **Which of the following statements comes closest to your view?**

- ○ The economic system in this country unfairly favors powerful interests

vs.

- ○ The economic system in this country is generally fair to most Americans

[q22] **Which of the following statements comes closest to your view?**

- ○ The obstacles that once made it harder for women than men to get ahead are now largely gone

vs.

- ○ There are still significant obstacles that make it harder for women to get ahead than men

6 Specific identities

The following list contains various words that people might use to describe their views.

[q23] Which of these words would you say apply to you?

Feel free to choose as many words as you like.

☐ Alt-right ☐ America First ☐ Anarchist ☐ Anti-racist ☐ Anti-vaccine ☐ Communist ☐ Democrat ☐ Environmentalist ☐ Feminist
☐ Independent ☐ Judeo-Christian Values ☐ Libertarian ☐ Moderate ☐ Nationalist ☐ Patriot ☐ Pro-life ☐ Progressive ☐ Republican
☐ Socialist ☐ Tea Party
☐ Other (specify) ______

7 Specific anti

[q24] Next, which of these would you say you are <u>opposed</u> to?

Feel free to choose as many words as you like.

☐ Alt-right ☐ America First ☐ Anarchist ☐ Anti-racist ☐ Anti-vaccine ☐ Communist ☐ Democrat ☐ Environmentalist ☐ Feminist
☐ Independent ☐ Judeo-Christian Values ☐ Libertarian ☐ Moderate ☐ Nationalist ☐ Patriot ☐ Pro-life ☐ Progressive ☐ Republican
☐ Socialist ☐ Tea Party
☐ Other (specify) ______

8 General identity questions

[q25] In general, would you describe your political views as...

- ○ Very liberal
- ○ Liberal
- ○ Moderate
- ○ Conservative
- ○ Very conservative

[q26] In politics today, do you consider yourself a...

- ○ Democrat
- ○ Republican
- ○ Independent
- ○ Other (specify) ______

9 Strength id

[q27] How strongly do you consider being a [political party] to be part of your core identity, of who you are?

- ○ Not at all
- ○ A little bit
- ○ A moderate amount
- ○ A large amount

[q28] Relative to mainstream political views in the US, to what extent do you consider yourself radical?

- ○ Not at all radical
- ○ Somewhat radical
- ○ Radical
- ○ Very radical

10 Own probability

[q29] What is the percent chance that you will vote in the upcoming 2020 U.S. Presidential election?

Please enter a number from 0 to 100, where 0 means that you will definitely not vote, and 100 that you will definitely vote. If you have already voted, please ente

______ %

12.1 Own voting intentions

[q30] If you do vote in the election, what is the percent chance you will vote for each of the candidates below?

For each candidate, please enter a number from 0 to 100, where 0 means that you will definitely not vote for that candidate, and 100 that you will definitely vote

Joe Biden (Democrat) ______

Donald Trump (Republican) ______

Someone else ______

13 Social probability

Now we would like you to think of your friends, family, colleagues, and other acquaintances who live in your state, are at least 18 years of age, and who you have communicated with at least briefly within the last month, either face-to-face, or otherwise. We will call these people your social contacts.

[q31] What percentage of your social contacts that live in your state are likely to vote in the upcoming 2020 U.S. Presidential election?

Please enter a number from 0 to 100, where 0 means that you think none of your social contacts will vote, and 100 means that all of your social contacts will vote

______ %

14.1 Social circle voting intentions

[q32] Out of all your social contacts who live in your state and are likely to vote in the 2020 U.S. Presidential election, what percentage do you think will vote for each of the candidates below?

For each candidate, please enter a number from 0 to 100, where 0 means that none of your social contacts will vote for that candidate, and 100 means that all of

Joe Biden (Democrat) ______

Donald Trump (Republican) ______

Someone else ______

15 Demo

Before we end, we have a few questions about yourself.

[q33] What is your age?

______ years

[q34] What is your gender?

- ○ Male
- ○ Female
- ○ Other

[q35] What is your highest level of formal education?

- ○ Did not finish high school
- ○ High school diploma
- ○ Some college
- ○ Bachelor's degree
- ○ Graduate degree

[q36] What is your race and/or ethnicity?

Please check all that apply

- ☐ White
- ☐ Black or African American
- ☐ Hispanic, Latino, or Spanish origin
- ☐ American Indian or Alaska Native
- ☐ Asian
- ☐ Native Hawaiian or Other Pacific Islander
- ☐ Some other race or ethnicity: ______

Table S3: Far-left and far-right favorite news source

Far-left	Far-right
Jacobin	Breitbart
Vox	Infowars
The Young Turks	Twitchy
Chapo	RedState
DailyKos	Gateway Pyndit
Current Affairs	Daily Caller
	4chan
	The Blaze
	8kun
	Daily Wire
	Daily Mail
	Citizen Free Press
	Conservative Treehouse

Table S4: Farl left and far-right identities

Far-left	Far-right
Anarchist	Alt-Right
Communist	Tea Party
Socialist	Confederate
	Identitarian
	Proud Boys

Table S5: Far-left and far-right mouvements

Far-left	Far-right
BLM	Unite the Right
Antifa	Stormer Book Club
Anonymous	League of the South
Indivisible	Traditionalist Worker Party
Occupy	Vanguard America
Communist Party USA	Loyal White Knights
Democratic Socialists of America	Fraternal Order of Alt-Knights
Workers World Party	American Identitarian
American Party of Labor	Identity Exropa

3 Rating and classification of of tweets

Online participants answered four questions for 5,215 tweets total (Figure S3). Of those, 4,752 received enough ratings to be evaluated according to various criteria (Table S6). A total of 164 tweets were pre-selected as covert and overt tweets based on these criteria.

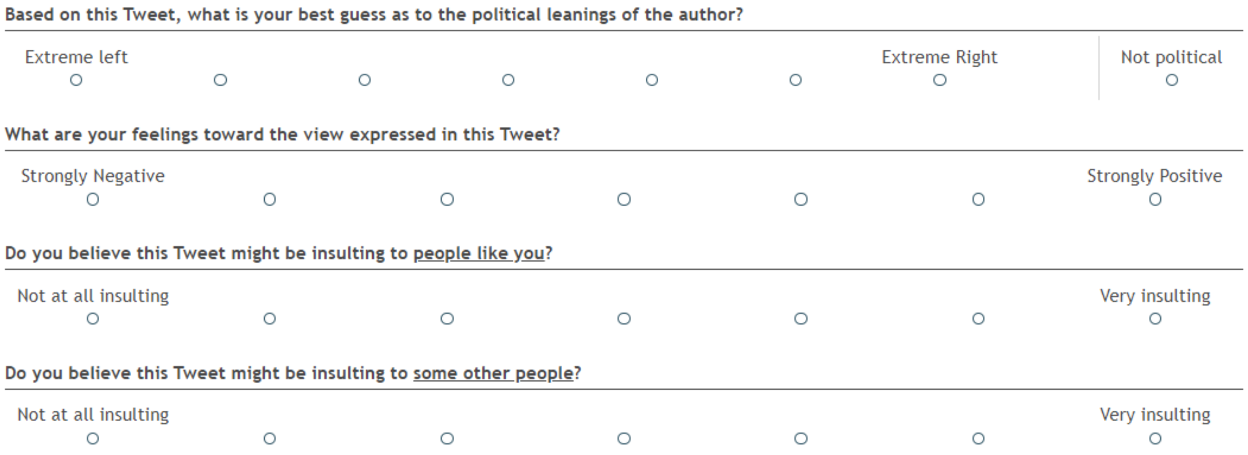
Based on this Tweet, what is your best guess as to the political leanings of the author?

Extreme left — Extreme Right | Not political

What are your feelings toward the view expressed in this Tweet?

Strongly Negative — Strongly Positive

Do you believe this Tweet might be insulting to people like you?

Not at all insulting — Very insulting

Do you believe this Tweet might be insulting to some other people?

Not at all insulting — Very insulting

Figure S3: Online questionnaire for rating tweets

3.1 Tweet descriptives

Figure S4 shows the mean tweet-level scores for each of the items within our two dimensions used to automatically pre-select tweets, separately for four different groups of raters defined in relationship to the political orientation of the tweet: copartisan far-left/right raters, copartisan moderate raters, cross-partisan far-left/right raters and cross-partisan moderate raters (for a total of n=1,992 raters). For example, in the case of a right leaning tweet, copartisans would be right-leaning raters and cross-partisan left-leaning. Of all tweets, 93 were marked as overtly political and 71 as covertly political. As described above, each tweet was given a

Table S6: Criteria used to select covert and overt tweets for the behavioral experiment, based on ratings given by different political groups

**Criteria for left (right) covert tweets**	
Copartisan difference in tweets' perceived political orientation	Difference between average political orientation ratings* given by far-and moderate left (right) raters is above 80th percentile.
Copartisan difference in affective response	Difference between average negative affect* experienced by moderate and far-left (right) raters is above 80th percentile.
Neutrality of moderates-rated political orientation	Average rating of political orientation given by left (right) moderates is neutral or mildly left (right).
Moderates' affective response	Average negative affect experienced by left (right) moderates is low (<=4 on a 7-point scale).
Political content	At least 75% of far-left (right) raters think the tweet is political.
**Criteria for left (right) overt tweets**	
Cross-partisan difference in political orientation	Difference between average political orientation ratings* given by left and right raters is below 20th percentile.
Cross-partisan difference in affective response.	Difference between average negative affect* experienced by left and right raters is above 80th percentile.
Copartisan similarity in affective response	Difference between average negative affect* experienced by far-and moderate left (right) raters is below 20th percentile.
Political content	At least 75% of all raters think the tweet is political.

*To make differences comparable across tweets, differences were divided by the sum of standard deviations of ratings given by each group of raters.

score based on the average ratings for each of the four groups, Figure S4 presents the mean of the scores across all tweets.

According to the definition of covert signaling, all raters should receive the political signal of overt tweets but only ingroup (copartisan) far-left/right raters should receive the political signal of covert tweets. This is reflected in the first two rows of Figure S4. On the one hand, overt tweets are rarely viewed as not political across all four groups (orange bar in the first row Figure S4). On the other hand, covert tweets are rarely selected as non political by copartisan far-left/right raters but quite more often by the three other groups (grey bar in the first row Figure S4). Regarding guessing the political identity of the tweet author, overt tweets were considered as extreme left or right for all groups, but covert tweets were not considered as extreme by copartisan moderate raters compared to copartisan far left/right raters (grey bar in the second row, first two columns in Figure S4). Cross-partisan raters considered covert tweets still as pretty politically extreme. We believe this is due to the opposite political side being more sensitive to any signal from the other political group, even if they do not quite understand it (and tend to mark it as non political). Both cross-partisan groups, however, still marked it as less politically extreme than copartisan far-left/right raters.

As expected, cross-partisan raters disliked all tweets from the opposite side of the political spectrum, but disliked overt tweets more than covert tweets. Copartisan raters did not view selected overt and covert tweets negatively (third row, first two columns in Figure S4), nor did they view them as insulting to people like them (fourth row first two columns in Figure S4). As expected, copartisan far-left/right raters were more positive and viewed the tweets as less insulting compared to copartisan moderate raters. However, for copartisan groups, there was no difference in the perception of overt or covert tweets. The difference in affective response between covert and overt tweets only appears within the ratings of cross-partisan members (last two columns of the last two rows of Figure S4). Members of the opposite political side viewed selected overt tweets more negatively and more insulting to their group compared to covert tweets. These differences in average ratings across measures give some evidence that the pre-selected tweets represented overt and covert political signaling at the time of the 2020 presidential elections. We further test this by comparing their usage on Twitter in the next Supplementary section.

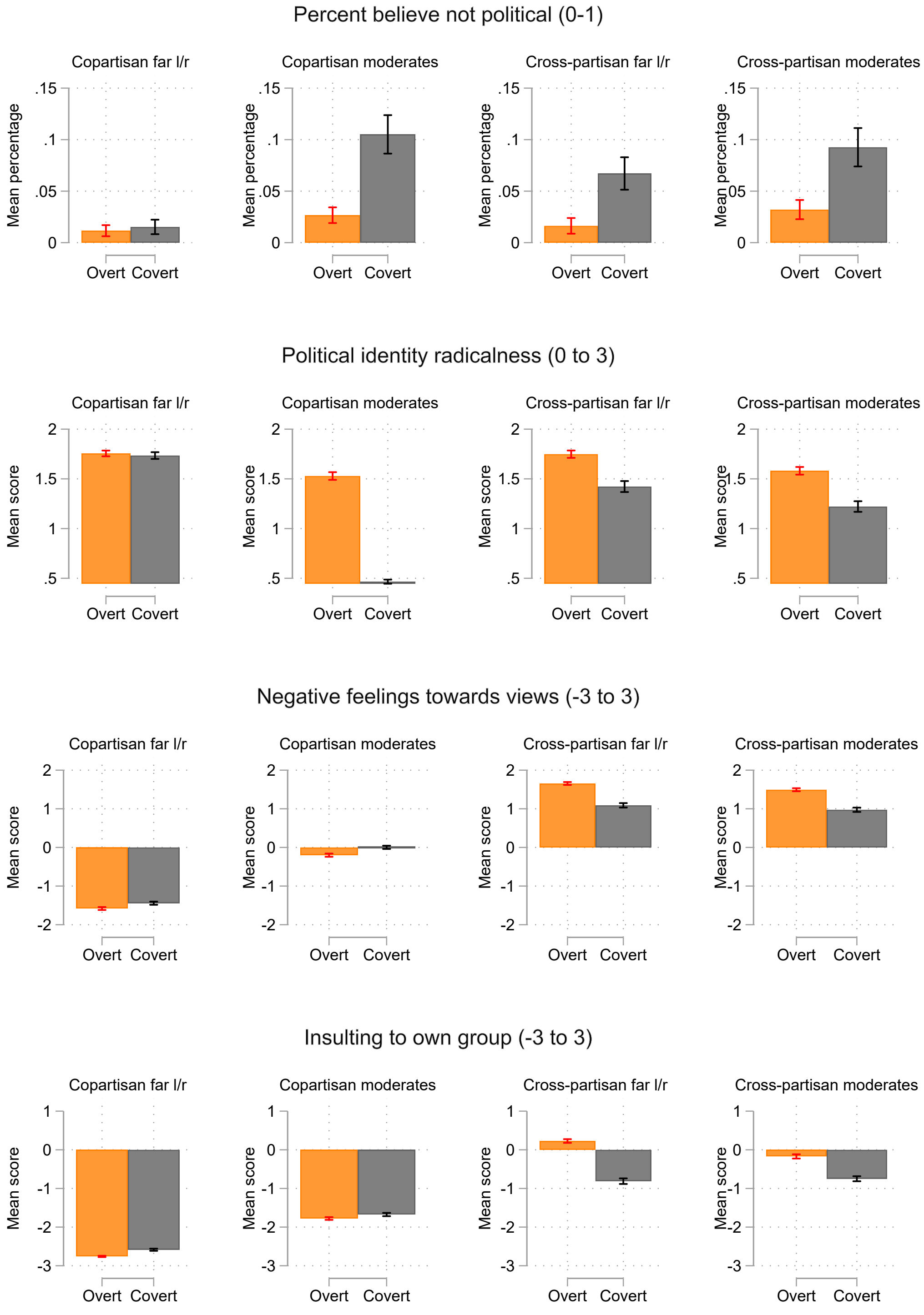

Figure S4: Mean tweet-level scores on four items from copartisan, cross-partisan, moderate, and far-left/right raters, $n = 164$. Error bars represent standard errors of the mean.

3.2 Description of covert and overt tweets' networks

One of the predictions of the general theory of covert signaling is that covert signaling should be more prevalent among individuals in more heterogeneous communities. To explore if this was true for our pre-selected tweets in a real world context, we compared the Twitter networks (heterogeneous vs. homogeneous) of the Twitter accounts for the full sample of tweets pre-selected as covert and overt (n = 164). As shown in Figure S5, both overt and covert political tweets were more likely to be from Twitter accounts with homogeneous followers compared to heterogeneous followers (61% of overt tweets were in heterogeneous contexts and 58% of covert tweets). Covert tweets were more likely than overt tweets to appear in a heterogeneous context: 42% of covert tweets were used in heterogeneous contexts compared to 39% of overt tweets. In other words, all political speech is more likely to happen in homogeneous Twitter networks compared to heterogeneous networks, but when political speech happens in heterogeneous networks it is more likely to be covert. These results are in line with the theory of covert signaling and point to the reasonability of our measures.

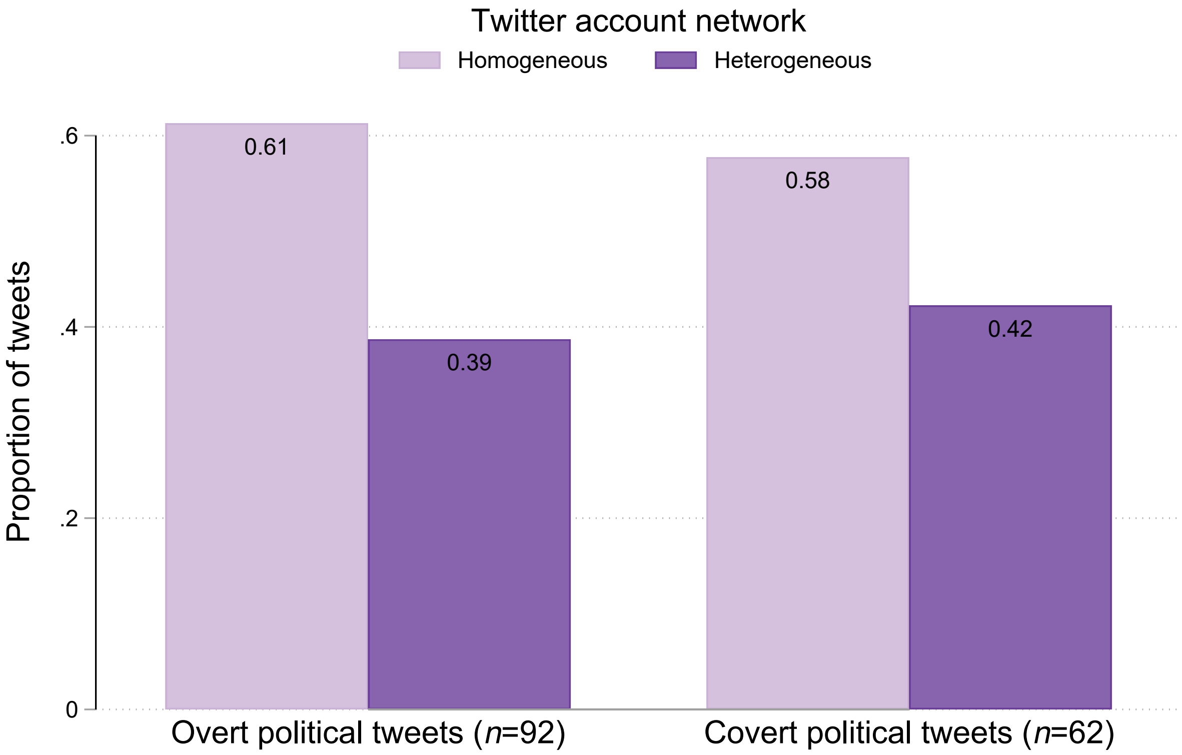

Figure S5: Proportion of overt and covert tweets in each type of Twitter account network.

Although the difference in the relative use of covert signaling in homogeneous and heterogeneous networks on Twitter was in the predicted direction, it was relatively small. This is reasonable, given the small sample of selected covert and overt tweets as well as the vast number of factors that influence online communication in a public forum such as Twitter. We hope future research pursues this line of inquiry and develops a wider data collection

process and measures of covertness to formally compare the use of covert political identity signaling on Twitter.

4 Behavioral Experiment

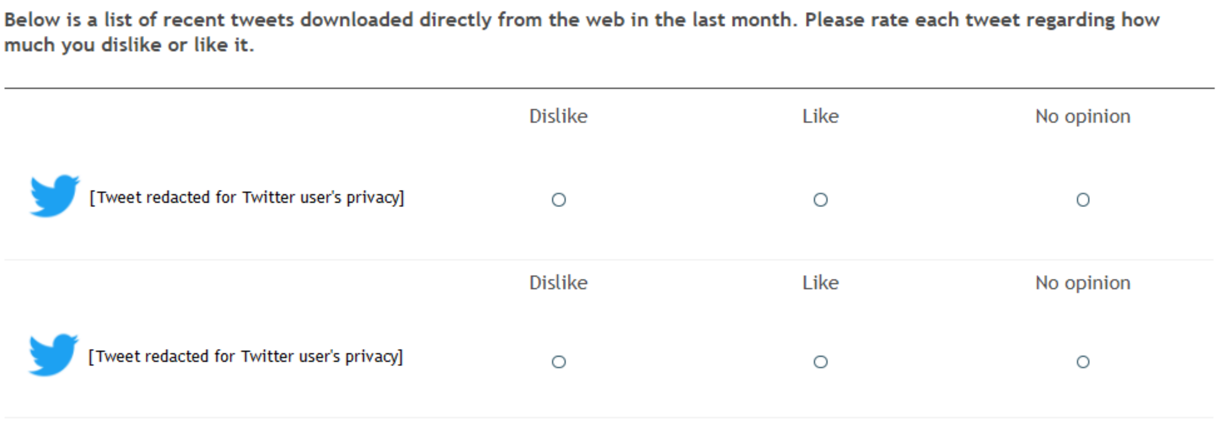
Below is a list of recent tweets downloaded directly from the web in the last month. Please rate each tweet regarding how much you dislike or like it.

	Dislike	Like	No opinion
[Tweet redacted for Twitter user's privacy]	○	○	○

	Dislike	Like	No opinion
[Tweet redacted for Twitter user's privacy]	○	○	○

Figure S6: First part of experiment: rating of each of the 80 tweets selected as covert and overt from both co- and cross-partisan sources.

Cross-partisan audience (*n*=243)

PLEASE READ THE INSTRUCTIONS CAREFULLY, THE TEXT IN RED WILL CHANGE FOR EACH PAGE.

You are now interacting with **ten other people** who are also participating in this study.

[Size group 1] described themselves as being **on the left of** the political spectrum, and **[size group 2]** described themselves as being **on the right.**

For **each person who likes** most of the tweets you share, you will receive an **additional 1 cent**.
For **each person who dislikes** most of the tweets you share, you will **lose [cost of dislike]**.

In other words, **for this page**, if all ten people like most of the tweets you share, you will receive **additional 10 cents**. If all ten people dislike most of the tweets you share, you will **lose [total possible cost]**.

Copartisan audience (*n*=235)

PLEASE READ THE INSTRUCTIONS CAREFULLY, THE TEXT IN RED WILL CHANGE FOR EACH PAGE.

You are now interacting with **ten other people** who are also participating in this study.

[Size group 1] described themselves as being **on the far left (right) of** the political spectrum, and **[Size group 2]** described themselves as being **mainstream left (right).**

For **each person who likes** most of the tweets you share, you will receive an **additional 1 cent**.
For **each person who dislikes** most of the tweets you share, you will **lose [cost of dislike]**.

In other words, **for this page**, if all ten people like most of the tweets you share, you will receive **additional 10 cents**. If all ten people dislike most of the tweets you share, you will **[total possible cost]**.

Each participant receives 8 different combinations of group sizes and costs of dislike:

1) Size group 1 (and group 2): 1(9), 4(6), 6(4), 9(1)
2) Cost of dislike (and total possible cost): 0.5 cent each (5 cents total) or 1 cent each (10 cents total).

Figure S7: Second part of experiment: material and conditions. Text in red brackets was replaced by different values for group sizes and costs of dislikes listed in the bottom box.

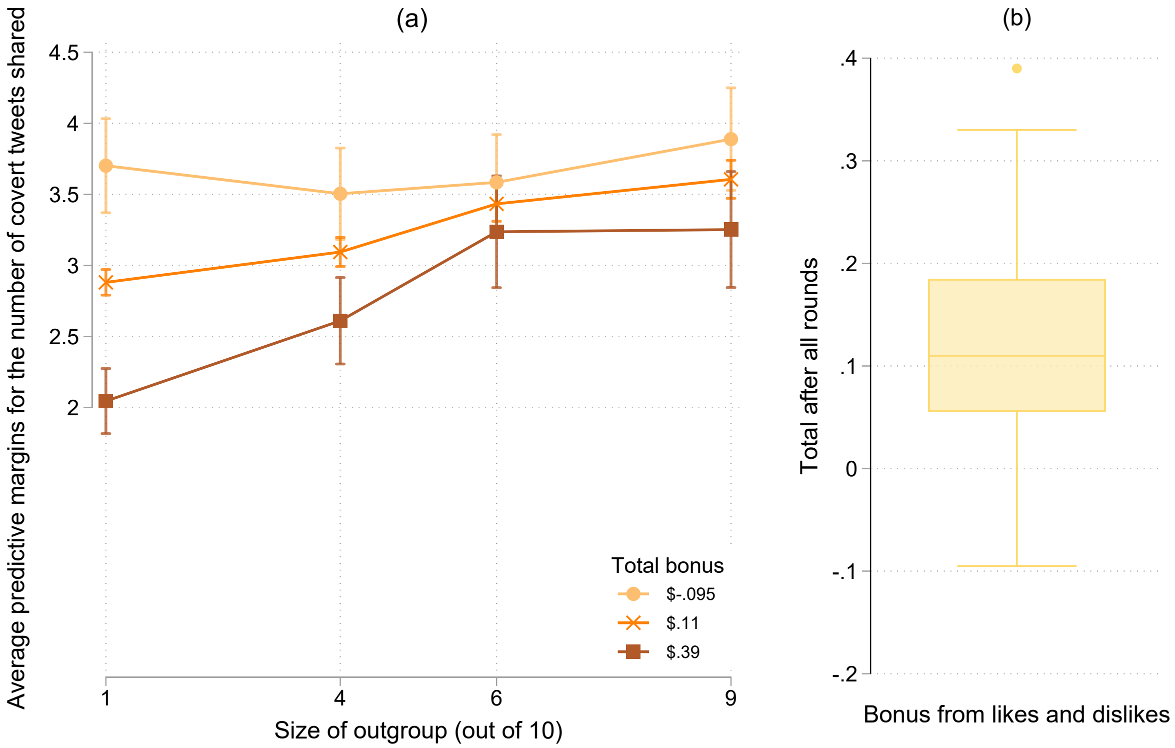

Figure S8: Bonuses received at the end of the behavioral experiment for participants with cross-partisan audiences, n=243. Average predictive margins for the use of covert tweets with 95% confidence intervals by total payoff (a) and sample distribution of total bonuses received at the end of the eight experimental rounds (b). Estimates are from multilevel Poisson models with random intercepts by individuals over experimental conditions, averaging predicted number of tweets shared across sample values for number of tweets shared, political leaning, age, gender, race, and education.

4.1 Power analysis

For the behavioral experiment, we used two independent samples (copartisan and cross-partisan comparisons) and four groups of raters (far left, moderate left, moderate right, and far right). With our budget and time to share tweets, we estimated that we could get 60 individuals per category (a total of 480 participants).

We calculated the power for detecting a linear increase in the total number of covert tweets shared over the outgroup size with one predictor (comparison), another within effect (cost), two independent samples (cross-partisan raters and copartisan raters), and controlling for the total number of tweets shared, for a total sample size of 480 participants.

For the cross-partisan high-cost condition, we assumed a conservative increase in the average number of covert tweets shared for each additional outgroup audience member, from zero covert tweets when there are no outgroup members to one covert tweet when there are only outgroup members (see estimates for our actual outgroup sizes in Table S7). We also assumed that for the low-cost condition (where dislikes were half as costly), the increase would be a third less than in the high-cost condition. We also assumed that participants in the copartisan condition would generally share half of the number of covert tweets compared to the cross-partisan condition, but the effect of outgroup size would not be different than in the cross-partisan condition.

We assumed the standard deviation for the total number of covert tweets shared would be around two tweets and we assumed the standard deviation would increase over outgroup size and be higher for the low-cost option. We expected the total number of tweets to have a standard deviation of eight and to be highly correlated with the total number of covert tweets shared, although decreasing over outgroup size.

Table S7: Estimated effect sizes across experimental conditions

	**cross-partisan**		**copartisan**	
**outgroup size**	high-cost	low-cost	high-cost	low-cost
**1**	0.10	0.10	0.05	0.05
**4**	0.40	0.33	0.20	0.16
**6**	0.60	0.48	0.30	0.24
**9**	0.90	0.70	0.45	0.35

Using the statistical software GLIMMPSE [1], we used the LEAR model to describe correlations which monotonically decrease with distance between the outgroups and costs. Using the Hotelling Lawley Trace test, we find that we have at least a 96.2% chance of detecting the effect of outgroup size with a sample size of 60 per group with a 0.01 error rate. For the same sample and error rate, we have a 99.4% chance of detecting an interaction between cost and outgroup size.

4.2 Sample descriptives

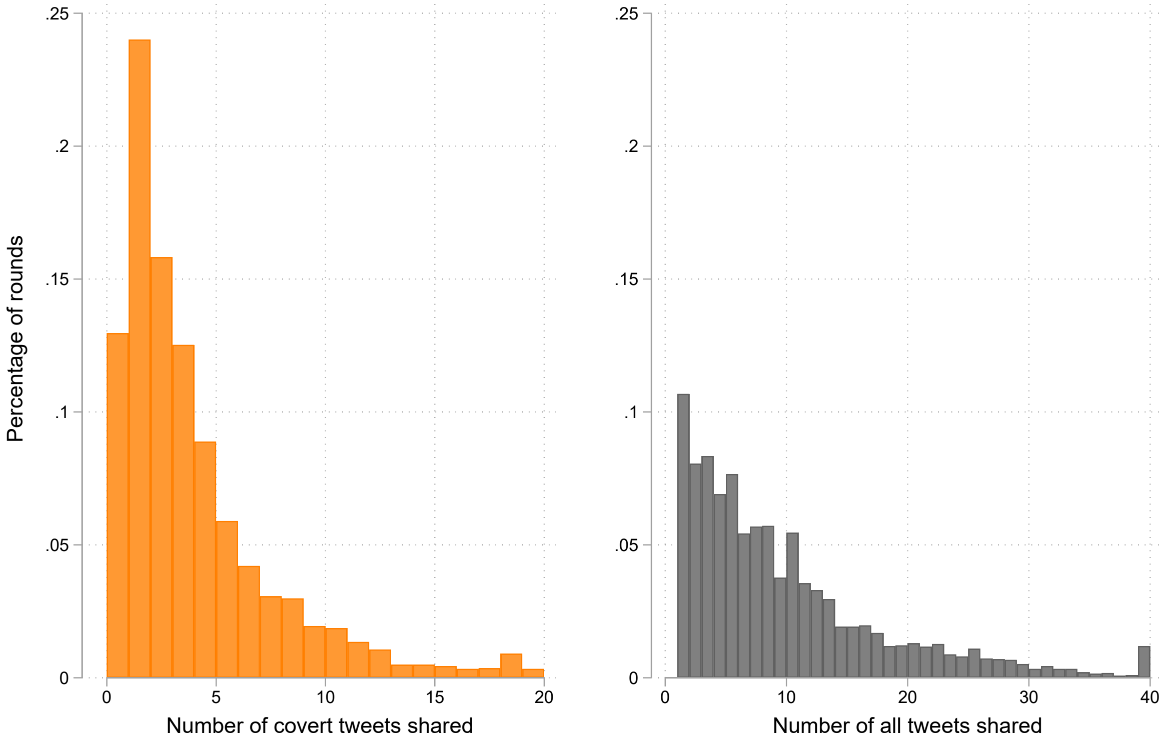

Figure S9: Proportion of rounds by the number of covert and total tweets shared.

Table S8: Sample Descriptive Statistics (N = 481)

	Far left		Mainstream left		Mainstream right		Far right	
	Mean/Prop.	SD	Mean/Prop.	SD	Mean/Prop.	SD	Mean/Prop.	SD
Age	34.14	10.90	42.21	13.32	40.02	10.44	44.66	13.38
Women	.51		.43		.59		.32	
Non-white	.23		.18		.12		.09	
College educated	.63		.59		.57		.62	

4.3 Statistical model

To compare the effect of different experimental conditions and individual-level characteristics, we conducted a generalized mixed-effects Poisson count model on the number of covert tweets shared per round with each outgroup experimental condition nested within individuals. An analysis of the linear mixed-effects model showed enough variation between individuals to warrant random intercepts for each individual (intra-class correlation between 0.304 and 0.775 across models and significant Breusch-Pagan test for cluster-level variation ($583 < \chi^2 < 3973$). Given that we want to analyze the number of all tweets and covert tweets shared, we approximated our outcome distribution using a Poisson distribution. We compared the addition of outgroup size as a continuous or categorical variable and found the latter to be a better fit. The resulting point estimates are in Table S9.

Table S9: Poisson mixed model estimates on count of covert (a and b) and total (c and d) tweets shared in each round in the copartisan (a and c) and cross-partisan (b and d) conditions. Estimates used for Figure 5. Standard errors in parenthesis.

	**(a) n=238**	**(b) n=243**	**(c) n=238**	**(d) n=243**
**Size of outgroup**				
(ref=1 out of 10)				
4 out of 10	0.024	0.063	-0.031	-0.203
	(0.048)	(0.049)	(0.028)	(0.029)
6 out of 10	0.007	0.139	-0.040	-0.495
	(0.048)	(0.052)	(0.028)	(0.032)
9 out of 10	0.030	0.183	-0.001	-0.627
	(0.047)	(0.053)	(0.027)	(0.033)
**High cost (ref=low cost)**	0.036	-0.041	-0.037	0.028
	(0.048)	(0.046)	(0.028)	(0.028)
**Size of outgroup * cost**				
(ref= 1 out of 10 and low cost)				
4 out of 10 & high cost	-0.009	0.026	0.029	-0.073
	(0.068)	(0.068)	(0.039)	(0.041)
6 out of 10 & high cost	-0.014	0.080	0.042	-0.033
	(0.068)	(0.071)	(0.039)	(0.045)
9 out of 10 & high cost	-0.062	0.088	0.029	-0.080
	(0.067)	(0.072)	(0.039)	(0.047)
**Total number of tweets shared**	0.081	0.079		
	(0.002)	(0.002)		
**Right participants (ref=left participants)**	0.013	-0.005	-0.048	-0.085
	(0.054)	(0.048)	(0.098)	(0.097)
**Individual-level controls**				
Age	0.001	-0.004	0.002	0.002
	(0.002)	(0.002)	(0.004)	(0.004)
Women (ref=men)	-0.129	-0.053	-0.125	-0.078
	(0.054)	(0.048)	(0.097)	(0.095)
White (ref=non-white)	-0.025	0.022	0.157	0.108
	(0.080)	(0.063)	(0.146)	(0.126)
College educated (ref=no college)	0.045	-0.009	-0.074	-0.227
	(0.055)	(0.048)	(0.100)	(0.096)
Constant	0.016	0.323	2.218	2.238
	(0.116)	(0.098)	(0.196)	(0.180)
Individual-level variance	0.116	0.084	0.529	0.513
	(0.018)	(0.013)	(0.051)	(0.050)

In Table S10 we present the linear mixed model of the number of covert tweets shared in copartisan and cross-partisan conditions centered by group. The main results are sub-

Table S10: Linear mixed model estimates on count of covert tweets shared in each round in the copartisan (a) and cross-partisan (b) conditions with covariance matrices centered by group. Standard error in parenthesis.

	**(a) n=238**	**(b) n=253**
**Size of outgroup**		
(ref=1 out of 10)		
4 out of 10	0.007	0.320
	(0.093)	(0.103)
6 out of 10	0.021	0.658
	(0.093)	(0.104)
9 out of 10	0.078	0.869
	(0.093)	(0.105)
**High cost (ref=low-cost)**	0.112	-0.007
	(0.093)	(0.103)
**Size of outgroup * cost**		
(ref= 1 out of 10 and low-cost)		
4 out of 10 & high-cost	-0.060	-0.002
	(0.131)	(0.145)
6 out of 10 & high-cost	-0.064	0.039
	(0.131)	(0.145)
9 out of 10 & high-cost	-0.195	0.118
	(0.131)	(0.145)
**Total number of tweets shared**	0.423	0.407
	(0.005)	(0.005)
**Right participants (ref=left participants)**	0.305*	-0.067
	(0.142)	(0.112)
**Individual-level controls**		
Age	0.003	-0.008
	(0.006)	(0.004)
Women (ref=men)	-0.406	-0.239
	(0.141)	(0.110)
White (ref=non-white)	0.168	-0.223
	(0.213)	(0.146)
College educated (ref=no college)	0.326	0.190
	(0.146)	(0.111)
Constant	-1.227	-0.081
	(0.296)	(0.223)
**Individual-level variance**	1.031	0.559
	(0.053)	(0.033)
**Group-level variance**		
Far left	1.088	1.058
	(0.039)	(0.036)
Mainstream left	1.025	1.422
	(0.050)	(0.048)
Mainstream right	0.887	1.632
	(0.049)	(0.049)
Far right	1.170	1.1775
	(0.051)	(0.048)

stantively similar to those in Table S9. However, the linear models enabled the estimation of different covariance matrices for each political group. The variance of the residuals shows that in the copartisan condition (a), participants from the far left and far right had a larger variance than those in the mainstream. However, in the cross-partisan condition (b), participants from the far left and far right had lower variance than those who were more mainstream.

References

[1] Kreidler, S., Muller, K., Grunwald, G., Ringham, B., Coker-Dukowitz, Z., Sakhadeo, U., Barón, A., and DH, D. G. (2013). Glimmpse: online power computation for linear models with and without a baseline covariate. *Journal of Statistical Software*, 54(10).

[2] Levay, K. E., Freese, J., and Druckman, J. N. (2016). The demographic and political composition of mechanical turk samples. *Sage Open*, 6(1):1–17.
